# Rhizosphere microbial shifts drive amygdalin detoxification and jasmonate-mediated alleviation of peach autotoxicity

**DOI:** 10.1093/ismejo/wrag095

**Published:** 2026-04-16

**Authors:** Jinzhi Yang, Haowei Du, Fan Tao, Muhammad Atiq Ashraf, Xusheng Gao, Xue Huang, Kaijie Zhu, Guohuai Li, Jinshui Zheng, Paola Bonfante, Francesca Cardinale, Junwei Liu

**Affiliations:** National Key Laboratory for Germplasm Innovation and Utilization of Horticultural Crops, College of Horticulture and Forestry Sciences, Huazhong Agricultural University, Wuhan, Hubei 430070, China; National Key Laboratory for Germplasm Innovation and Utilization of Horticultural Crops, College of Horticulture and Forestry Sciences, Huazhong Agricultural University, Wuhan, Hubei 430070, China; National Key Laboratory for Germplasm Innovation and Utilization of Horticultural Crops, College of Horticulture and Forestry Sciences, Huazhong Agricultural University, Wuhan, Hubei 430070, China; National Key Laboratory for Germplasm Innovation and Utilization of Horticultural Crops, College of Horticulture and Forestry Sciences, Huazhong Agricultural University, Wuhan, Hubei 430070, China; National Key Laboratory for Germplasm Innovation and Utilization of Horticultural Crops, College of Horticulture and Forestry Sciences, Huazhong Agricultural University, Wuhan, Hubei 430070, China; National Key Laboratory for Germplasm Innovation and Utilization of Horticultural Crops, College of Horticulture and Forestry Sciences, Huazhong Agricultural University, Wuhan, Hubei 430070, China; National Key Laboratory for Germplasm Innovation and Utilization of Horticultural Crops, College of Horticulture and Forestry Sciences, Huazhong Agricultural University, Wuhan, Hubei 430070, China; National Key Laboratory for Germplasm Innovation and Utilization of Horticultural Crops, College of Horticulture and Forestry Sciences, Huazhong Agricultural University, Wuhan, Hubei 430070, China; National Key Laboratory of Agricultural Microbiology, College of Informatics, Huazhong Agricultural University, Wuhan, Hubei 430070, China; Department of Life Sciences and Systems Biology, Turin University, Torino 10125, Italy; PlantStressLab, Department of Agriculture, Forestry and Food Sciences (DISAFA), Turin University, Grugliasco, Torino 10095, Italy; National Key Laboratory for Germplasm Innovation and Utilization of Horticultural Crops, College of Horticulture and Forestry Sciences, Huazhong Agricultural University, Wuhan, Hubei 430070, China

**Keywords:** autotoxicity, jasmonic acid, microbial assembly, perennial plant, *Prunus persica*, soil–plant feedback

## Abstract

Plant-associated microbes play essential roles in maintaining plant health and modulating responses to environmental stresses. Autotoxicity from allelopathic compounds is a major constraint on perennial crop production, yet the potential for plants to recruit microbiota to counteract such toxicity remains understudied. Our research combined field sampling from a multi-replant peach system, multi-omics, pot, and hydroponic experiments to elucidate plant–microbe interactions that alleviate amygdalin-induced autotoxicity. Metabolomic analysis of peach orchard soils showed that amygdalin accumulated progressively in the rhizosphere with longer continuous cultivation. Exogenous amygdalin inhibited plant growth, with stronger suppression in sterilized soil, suggesting a protective role of soil microbes. Amygdalin application altered rhizobacterial community structure and enriched several taxa, including *Burkholderia-Caballeronia-Paraburkholderia* and *Sinomonas*. *In vitro* assays confirmed that amygdalin serves as a selective substrate for these enriched bacteria. We further found that three strains isolated from the amygdalin-stressed peach rhizosphere significantly alleviated autotoxic inhibition, and their co-inoculation showed the greatest enhancement of plant performance. Metabolomic and transcriptomic analyses revealed activation of plant jasmonic acid (JA) pathway. Its involvement was confirmed by the alleviation of amygdalin-induced stress upon exogenous JA application and by the attenuation of microbiota-mediated stress relief upon JA pathway inhibition. Our study reveals a critical mechanism by which plants enrich specialized microbes that can alleviate autotoxicity by direct amygdalin degradation, activation of the JA pathway, and modulation of redox homeostasis in peach. These findings provide new insights into plant–microbe interactions in perennial systems and highlight the potential of microbiome-informed microbial interventions for mitigating replant disease.

## Introduction

Plant–microbe interactions fundamentally shape plant evolution, ecology, and productivity. Plant-associated microbial communities, which act as integrated microbial consortia, enhance plant fitness through hormone regulation [1], nutrient mobilization [2], and pathogen suppression [3]. Thus, understanding these interactions is essential for developing microbiome-based strategies to improve soil health, enhance crop resilience, and promote sustainable agriculture. The multi-decade lifespan of perennial crops fosters a stable and co-evolved rhizosphere microbiome, positioning them as a unique system for studying plant–microbe interactions [4]. This differs from annual crops, in which yearly ploughing, replanting, and rotation lead to strong microbial turnover [5–7]. Perennial plant root systems and litter continuously provide stable habitats and carbon inputs that sustain microbial populations [8]. These long-term associations are thought to play a crucial role in nutrient cycling and stress resistance, but despite being critical for ecosystems and agriculture, we still lack a clear understanding of how perennial plants shape and exploit their rhizosphere microbial communities.

Plant specialized metabolites, such as glucosinolates [9], terpenoids [10], flavonoids [11], and phenolic compounds [12], are central mediators of plant–environment interactions. Synthesized in response to diverse environmental stimuli, these metabolites function as biochemical mediators of adaptation and defense, substantially enhancing plant survival under stress [13, 14]. Among such metabolites, allelochemicals represent a class of bioactive secondary molecules that can inhibit the growth of competing plants after their release through root exudates, leaf and fruit leachates, and from decomposing residues. A recent meta-analysis shows that allelopathy is taxonomically widespread, with ~72% of plant families capable of producing such compounds [15].

Allelochemicals serve multiple ecological roles, including defense against herbivores and pathogens, and promotion of beneficial microbial associations. Their targeted action and biodegradability favor them as sustainable alternatives to synthetic agrochemicals, with promising applications in weed management and crop production [16, 17]. However, sustained release of root exudates, decomposition of plant residues, and reduced microbial degradation capacity contribute to the progressive buildup of allelochemicals. Excessive accumulation of these compounds often induces negative soil–plant feedback, resulting in inhibited growth, reduced yield, and impaired stress tolerance, and poses an even greater challenge in perennial replanting systems [18]. The decline in plant fitness is thought to arise not only from direct phytotoxicity, but also from indirect mechanisms such as reduced nutrient availability and dysbiosis of rhizosphere microbial communities [19]. Yet the specific roles and effect sizes of individual allelochemicals in these processes are underexplored.

Rather than passive victims, plants experiencing abiotic or biotic stress can enhance their resilience by shaping the rhizosphere to favor beneficial soil microbes. The resulting microbial communities perform functions that support plant stress resilience and overall fitness [20, 21]. Under allelopathic stress, these interactions also induce compositional and functional shifts in the rhizosphere microbiota, often affecting bacterial communities more strongly than fungal assemblies [22]. Allelochemicals serve as signaling molecules or energy sources, driving the enrichment of bacteria with specialized metabolic functions, such as detoxification. Several studies using exogenous additions of plant-derived allelochemicals have shown that these compounds select for microbial taxa that either directly degrade them or indirectly enhance plant fitness via growth promotion [23, 24]. However, despite these advances, the key microbial taxa and mechanisms underlying the mitigation of allelopathic autotoxicity in plants remain largely unknown.

Peach (*Prunus persica*), a globally important perennial fruit crop, is often constrained by the replant problem, a soil-borne disorder wherein newly planted young trees exhibit lower survival rates or severe growth stunting during the first 1–2 years after replanting. The disease is strongly linked to the presence of allelochemicals such as benzoic acid from decomposing root residues of preceding trees [25, 26]. In this study, we utilized peach orchards with three different replanting histories, making them an excellent tool to understand autotoxin accumulation associated with plant–soil legacy. We combined multi-omics analysis of their rhizosphere soils with controlled pot and hydroponic experiments to address the following questions: (i) Which rhizosphere metabolites contribute to negative soil–plant feedback and plant growth suppression in permanent peach systems? (ii) How do autotoxic allelochemicals reshape the compositional and functional potential of microbial communities in the rhizosphere? (iii) Which rhizobacterial taxa alleviate autotoxicity in peach, and through what biological mechanisms? We hypothesized that long-term allelochemical accumulation in repeatedly replanted peach orchards selectively enriches bacterial taxa capable of degrading key allelochemicals or enhancing plant resistance to them.

## Materials and methods

### Field sampling

**Figure 1:**
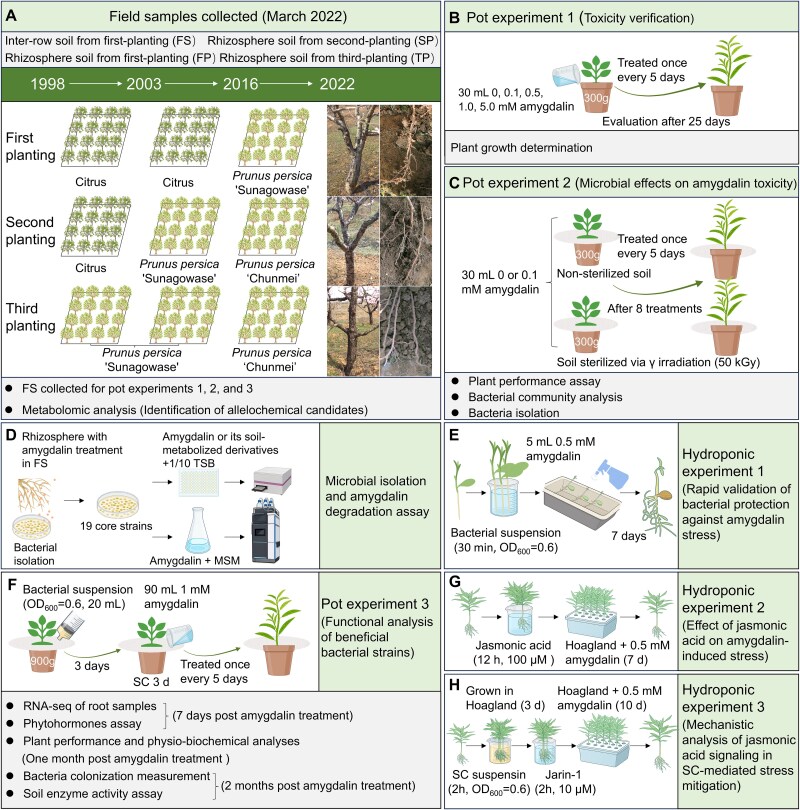
Schematic overview of the experimental design. (A) Field sampling strategy across peach orchards with different replant histories: (i) first planting (FP) of peach cultivar “Chunmei” following citrus cultivation (2016–2022); (ii) second-planting (SP) after one cycle of peach monoculture (“Sunagowase”, 2003–2016) prior to “Chunmei” planting (2016–2022); (iii) third-planting (TP) after two successive peach monocultures (“Sunagowase”, 1998–2003 and 2003–2016) before “Chunmei” planting (2016–2022). Bulk soil from the fallow soil (FS) of FP orchard without peach roots was collected as the control. (B) Pot experiment 1: Effects of amygdalin on peach seedling growth. (C) Pot experiment 2: Role of soil microbiota in alleviating amygdalin-induced stress using sterilized vs non-sterilized soils. (D) Bacterial community experiment, to test the effects of amygdalin and its soil-metabolized derivatives on bacterial growth, and the amygdalin-degrading ability of strains. MSM: minimal salt medium; TSB: tryptic soy broth medium. (E) Hydroponic experiment 1: Surface-sterilized peach seeds were inoculated with bacterial suspensions or sterile water, and treated with amygdalin (0.5 mM) or water to evaluate the ability of bacterial strains to alleviate amygdalin-induced stress. (F) Pot experiment 3: Peach seedlings were inoculated with three individual bacterial strains or their SC and exposed to amygdalin to investigate mechanisms of stress alleviation by the SC. (G) Hydroponic experiment 2: Peach seedlings were pretreated with JA before amygdalin exposure to assess its role in mitigating amygdalin-induced stress. (H) Hydroponic experiment 3: Peach seedlings were inoculated with SC for 2 h, transferred to half-strength Hoagland nutrient solution for 3 d, pretreated with 10 μM Jarin-1 (a JA-Ile biosynthesis inhibitor) for 2 h, and then cultured in half-strength Hoagland nutrient solution supplemented with 0.5 mM amygdalin for 10 d to evaluate the involvement of the JA pathway in SC-mediated alleviation of amygdalin-induced phytotoxicity.

In March 2022, soil samples were collected from a long-term peach orchard located in Xiaogan City, Hubei Province, China (30°59′ N, 114°5′ E). This site is characterized by a mean annual temperature of 14°C–22°C and average annual precipitation of 487 mm. Three field types representing different planting histories were selected (Fig. 1A). (i) A field at first planting of the peach cultivar “Chunmei” following Citrus cultivation was defined as first-planted (FP; from 2016 to 2022); (ii) A field with one cycle of peach monoculture (“Sunagowase”, 2003–2016) prior to “Chunmei” planting (2016–2022) was defined as second-planted (SP); (iii) A field with two successive peach monocultures (“Sunagowase”, 1998–2003 and 2003–2016) before “Chunmei” (2016–2022) was classified as third-planted (TP). Thus, the three plots mainly differ in the duration of continuous peach cultivation (6 vs 19 vs 24 years). All peach trees were grafted onto rootstock *P. persica* Batsch (“Maotao”). Additionally, bulk soil from the FP orchard without peach roots (fallow) was collected as control (FS). Rhizosphere soil was collected for metabolomic analysis following the procedure described in a previous study [27]. For each planting history, five plots were established. In each plot, three trees were randomly selected, and rhizosphere soil was collected from fine roots (Ø < 2 mm) located within a 50 cm radius around the trunk at a depth of 15–30 cm. All samples were collected with sterile tools, immediately frozen in liquid nitrogen, and stored at −80°C until further analysis.

### Growth chamber trials

Pot experiment 1 (Toxicity verification of amygdalin, Fig. 1B): To assess the effects of amygdalin on peach (*P. persica* Batsch, “Maotao”) growth, seeds were stratified in moist sand at 4°C for 12 weeks, germinated and transplanted to 32-hole trays containing a mixture of nutrient soil (Xingyuxing, China), peat moss (Pindstrup, Denmark), and vermiculite (Taoyang, China) at 3:1:1 (v:v:v). Uniform 10-day-old seedlings were transferred to plastic pots (Ø 8 cm bottom, Ø12 cm top, 10 cm height) filled with 300 g of soil collected from the FS before the field trial and maintained in a growth chamber (25°C, 65% relative humidity, 16 h:8 h light to dark cycle). After 5 days (d) of acclimation, seedlings were treated every 5 d with 30 ml of 0, 0.1, 0.5, 1.0, and 5.0 mM amygdalin (Sinopharm, China) solution, corresponding to soil concentrations of ~4.57, 22.85, 45.70, and 228.50 mg kg^−1^, respectively. Each treatment had three biological replicates, with four plants per replicate. After five treatment cycles, plants were harvested for morpho-physiological assessments.

Pot experiment 2 (Microbial effects on amygdalin toxicity, Fig. 1C): To assess the effect of microbial communities on amygdalin-induced stress, peach plants were prepared as above but in γ-irradiated (50 kGy, microbiota-depleted) or in non-sterilized FS. Seedlings received 30 ml of 0.1 mM amygdalin every 5 d (final soil concentration ≈ 4.57 mg kg^−1^). Control plants received an equal volume of sterile water. Soil moisture was maintained at ~60% water-holding capacity. After eight treatment cycles, plants were sampled for further analysis. For rhizosphere bacterial community analysis, rhizosphere soil from three seedlings per replicate (non-sterilized) was pooled into one composite sample, and four pooled replicates were prepared for sequencing.

Hydroponic experiment 1 (Rapid validation of bacterial protection against amygdalin stress, Fig. 1E): To quickly assess the impact of individual bacterial strains on peach root development under amygdalin-induced stress, surface-sterilized seeds with newly emerged white radicles were trimmed to retain only the primary root and immersed in bacterial suspension (OD_600_ = 0.6) or sterile water (Control) for 30 min. Three seedlings were placed in sterile plastic trays (28.5 × 19.5 × 7.0 cm) containing 200 ml sterile water with filter paper laid on the surface, sealed with transparent plastic film and placed in a growth chamber; each treatment was replicated with three trays. After 24 h, peach seedlings in each tray were sprayed with 5 ml of 0.5 mM amygdalin solution or sterile water (Control). Root morphology was evaluated one week later.

Pot experiment 3 (Functional analysis of beneficial bacterial strains, Fig. 1F): To assess the ability and mechanisms of isolated bacterial strains and their synthetic community (SC, see section below: “Culturable bacteria isolation and taxonomic analysis”), peach plants were grown in sterilized FS (900 g per pot, Ø11.0 cm bottom × Ø13.5 cm top × 12.5 cm height). The bacterial suspensions were prepared as follows: we first inoculated individually each of the isolates onto a tryptic soy broth (TSB) plate. After 3–5 d of incubation, a single colony was picked from each plate and incubated in liquid TSB by shaking at 180 rpm and 28°C for 3–5 d. After centrifugation and resuspension in sterile water, OD_600_ was adjusted to 0.8 and a 20 ml inoculum was applied near the roots. Starting 3 d later, seedlings were treated every 5 d with 90 ml of 1 mM amygdalin. Controls received an equal volume of sterile water each time. Each treatment was run in 20 biological replicates.

Hydroponic experiment 2 (Role of jasmonic acid in alleviating amygdalin stress, Fig. 1G): To examine whether JA enhances peach seedling tolerance to amygdalin-induced stress, morphologically uniform, 10-day-old seedlings were transplanted into plastic containers with 4 l of half-strength Hoagland solution and pre-cultured for 5 d. Subsequently, the seedlings were placed in solutions containing 0 or 100 μM JA for 12 h, then transferred to half-strength Hoagland solutions containing 0 or 0.5 mM amygdalin. Each treatment was performed with three independent biological replicates, and each replicate consisted of four individual plantlets arranged in one container. Nutrient solution was replaced every 3 d, and continuously aerated using air pumps, with dissolved oxygen maintained at 8.0–8.5 mg l^−1^ using an automated monitoring system (Leici, China). After 1 week, plants were harvested for morpho-biochemical analyses.

Hydroponic experiment 3 (Mechanistic analysis of JA signaling in SC-mediated stress mitigation, Fig. 1H): To further test whether JA signaling is required for SC-mediated stress alleviation, peach seedlings were inoculated by immersing their root systems for 2 h in a SC suspension, prepared by individually adjusting strains A7, P16 and C8 to OD_600_ = 0.6 and mixing them in equal volumes. After inoculation, seedlings were cultivated in half-strength Hoagland solution for 3 d. Roots were then treated with 10 μM Jarin-1 (jasmonoyl-isoleucine (JA-Ile) biosynthesis inhibitor; MedChemExpress, Cat # HY-115521, USA) for 2 h before transferring to fresh half-strength Hoagland solution supplemented with 0.5 mM amygdalin [28]. Each treatment comprised three biological replicates, and with 10 plantlets divided between two containers. Seedlings were cultivated for 10 d, with an additional application of Jarin-1 on Day 5 to reinforce the JA pathway inhibition.

### Metabolite profiling and quantification of cyanogenic glycosides and jasmonates

For metabolite profiling, 50 mg of freeze-dried field-collected soil samples (FS, FP, SP, TP from the experiment described in Fig. 1A) were placed in 2 ml centrifuge tubes with a grinding bead (Ø 6 mm) and extracted in 400 μL of 80% methanol containing 0.02 mg ml^−1^ of L-2-chlorophenylalanine (internal standard) by ultrasonic treatment (40 kHz) at 5°C for 30 min, followed by protein precipitation at −20°C for 30 min. After centrifugation at 4°C and 13 000 *g* for 15 min, supernatants were transferred to vials, and their metabolites were profiled using a Thermo UHPLC-Q Exactive HF-X system (Majorbio, Shanghai) equipped with an ACQUITY HSS T3 column (100.0 × 2.1 mm, 1.8 μm). The mobile phases were 0.1% formic acid in water/acetonitrile (95:5) and acetonitrile/isopropanol/water (47.5:47.5:5.0). The flow rate was 0.4 ml min^−1^, column temperature 40°C, and injection volume 3 μL.

For the quantification of cyanogenic glycosides, 2 g of soil (FS, FP, SP, TP from the experiment described in Fig. 1A) was extracted with 20 ml of 80% methanol, incubated at 65°C for 20 min, and then cooled on ice. The mixture was shaken overnight at 4°C and 150 rpm. After centrifugation at 10 000 *g* for 10 min, supernatants were filtered through a 0.45 μm polyvinylidene fluoride membrane. The extracts were dried under nitrogen flow, resuspended in 0.2 ml of 50% methanol, and stored at −80°C until analysis. Quantification was performed on an LC-10A HPLC system (Shimadzu, Kyoto, Japan) with a C18 column (4.6 × 250.0 mm, Thermo, USA) using methanol/water (20:80) at 0.7 ml min^−1^. Injection volume was 10 μL, and detection at 210 nm. Amygdalin (analytical standard) was purchased from Sigma–Aldrich (St. Louis, MO, USA), and prunasin was obtained from Tianzhi Biotechnology (Wuhan, China). The method achieved good chromatographic separation, and the calibration curve showed excellent linearity between peak area and analyte concentration, with *R*^2^ values of 1.00 for amygdalin and 0.99 for prunasin.

Jasmonates (JA and JA-Ile) were determined in roots following a previous method [29], with minor modifications. Briefly, 50 mg of freeze-dried tissue (from pot experiment 3, Fig. 1F) was ground in liquid nitrogen and extracted with 2 ml of cold isopropanol/water/HCl (1000:500:1, v:v:v) containing 1 μL of H_2_-JA (10 ng ml^−1^; Sigma, MO, USA). After shaking at 4°C, 150 rpm for 16 h, 4 ml of dichloromethane was added, followed by further shaking. Samples were centrifuged at 4°C and 6000 *g* for 15 min, ~4 ml of the lower phase was transferred to a tube, and dried under nitrogen flow. Residues were dissolved in 400 μL of methanol for 10 min, centrifuged (14 000 *g* for 20 min), dried again, and redissolved in 100 μL of 50% methanol. Total JA and JA-Ile were determined by electrospray ionization tandem mass spectrometry (UFLC-ESI-MS/MS; Shimadzu, Kyoto, Japan) analysis on three independent biological replicates per condition.

### 16S rRNA amplicon sequencing and analysis

The rhizosphere and endophytic compartment were isolated from the roots of peach following a previously described procedure [30]. The rhizosphere soil was washed away from roots by briefly vortexing them in sterile phosphate-buffered saline (PBS; pH = 7.4). After removing the roots, the suspensions were filtered through a 100 μm cell strainer and spun down for 10 min at 4000 *g*. The supernatants were discarded, and the remaining pellets were the rhizosphere soil samples. For the endosphere, the roots were washed twice more with sterile PBS, transferred to a new tube with sterile PBS, sonicated for 10 min at 60 Hz (output frequency 42 kHz, power 90 W, Zhixin Instrument, Shanghai, China) and dried on sterile filter paper. All samples were frozen in liquid nitrogen and stored at −80°C.

DNA was extracted from these two sets of samples using E.Z.N.A Soil DNA Kit (Omega Bio-tek), and DNA integrity and concentration were assessed via electrophoresis and NanoDrop spectrophotometry. Two primer pairs were used for 16S rRNA gene amplification: 338F and 806R for analyzing the effect of amygdalin on rhizosphere microbiota (from pot experiment 2, Fig. 1C), and 799F and 1193R for detecting the colonization of the SC in rhizosphere and root endophytic compartments [31] (from pot experiment 3, Fig. 1F; Table S1).

Amplicons were sequenced on a MiSeq System (Illumina) by Majorbio (Shanghai, China). Raw sequences were quality-filtered by FASTP v0.19.6 [32] and merged by FLASH v1.2.11 [33]. High-quality reads were denoised and clustered to amplicon sequence variants (ASVs) using the DADA2 plugin within the QIIME2 pipeline v2020.2 [34]. Taxonomic classification was performed with naïve Bayes classifier trained on the SILVA 16S rRNA v138 database. Alpha diversity was calculated in Mothur v1.30.1, and beta diversity was assessed by principal coordinate analysis (PCoA) based on Bray–Curtis dissimilarities using Vegan package. Linear discriminant analysis (LDA) effect size was used to identify the taxa with significantly varying abundance among treatments (LDA score > 3.5, *P* value <.05). Bacterial functions were predicted from ASVs on the 16S rRNA gene using PICRUSt2 [35].

### Culturable bacteria isolation and taxonomic analysis

Microorganism isolations from the rhizosphere were conducted in a previously described method, but with slight modifications [36]. Briefly, fresh rhizosphere soil (from pot experiment 2 with amygdalin-treated), 1 g non-sterilized FS was suspended in 9 ml sterile PBS, serially diluted (10^−2^ to 10^−7^), and plated onto selective media, including Reasoner’s 2A agar, TSB agar, and Gause’s synthetic agar. Plates were incubated at 28°C, and colonies were repeatedly streaked on TSB plates until pure cultures were obtained (Fig. 1D). Genomic DNA was extracted from purified isolates using the alkaline lysis method. The nearly full-length 16S rRNA gene was amplified using universal primers 27F and 1492R (Table S1), and sequenced (Tsingke, Beijing, China). Taxonomic identities were assigned using BLAST searches with default settings against the NCBI database. To link cultured isolates with in situ rhizosphere taxa identified by amplicon sequencing, the V3–V4 region of the 16S rRNA gene was re-amplified from isolates using primers 338F and 806R and aligned against representative ASVs from the rhizosphere dataset. Strains sharing ≥97% sequence identity to an ASV were considered matched. Phylogenetic relationships were inferred using the neighbor-joining method in MEGA v7.0. All bacterial isolates were cryopreserved at −80°C.

### Biodegradation and antibacterial activity of amygdalin

Amygdalin was dissolved in sterile water to prepare a 1 mM solution, which was then diluted 10 and 100-fold. For biodegradation assays (Fig. 1D), a peach rhizosphere soil suspension was obtained by suspending 1 g of soil from pot experiment 2 (amygdalin-treated, non-sterilized FS) in 100 ml of PBS. One ml of such suspension was added to 100 ml of 1 mM amygdalin (in 1:10 TSB), followed by incubation at 28°C with shaking at 180 rpm for 24, 48, and 72 h and by the analysis of degradation products.

The degradation efficiency of amygdalin by bacterial isolates was assessed by high-performance liquid chromatography (HPLC) following a previously established method [23]. A single colony of each isolate was inoculated into 50 ml of minimal salt medium (MSM) supplemented with amygdalin (50 mg l^−1^) and incubated at 28°C with shaking at 180 rpm for 5 d. Culture samples (100 μL) were collected at designated time points (0, 1, 2, 3, 4, and 5 d) and dried using a vacuum rotary evaporator. Residues were resuspended in 1 ml of 50% methanol, vortexed thoroughly, and filtered through 0.45 μm nylon membranes before HPLC analysis (Fig. 1D).

The antibacterial activities of amygdalin and of its degradation products against each isolate were evaluated by growth curve assays based on a previously reported method [37], with minor modifications. Briefly, a single colony of each strain was picked and inoculated into 1:2 TSB liquid medium:water, and shaken at 28°C until OD_600_ reached 1.0–2.0. The cultures were then diluted 100- to 500-fold with 1:10 TSB medium:water to standardize the initial bacterial density prior to growth assays. Growth curves of the isolates were defined in 96-well plates (Corning, flat bottom). Each well contained 90 μL of 1:10 TSB medium inoculated with the standardized bacterial suspension, supplemented with 10 μL of amygdalin at 1000, 100, 10, 0 μM, or with its degradation products collected after 0, 24, 48, and 72 h of incubation with the bacterial suspension, following the method described above. The OD_600_ values were monitored at 8, 16, 24, 32, 40, 48, and 56 h using a SpectraMax (Molecular Devices, USA) 96-well plate reader.

### RNA extraction, RNA-seq, and data analysis

Peach root samples (from pot experiment 3) with or without SC inoculation were collected after 7 d of amygdalin treatment in three biological replicates for RNA-seq analysis. Total RNA was extracted using TRIzol Reagent (Invitrogen, USA) following the manufacturer’s protocol. RNA quality and quantity were assessed using the 5300 Bioanalyzer (Agilent) and the ND-2000 (NanoDrop Technologies), respectively. A total of six RNA sequencing libraries were prepared from 1 μg of total RNA per sample using the Illumina Stranded mRNA Prep and ligation kit (Illumina, San Diego, USA). Clean reads were generated after raw data filtering, error rate correction, and GC content distribution analysis. These reads were then aligned to the *P. persica* reference genome (v2.0.a1) using TopHat2 (v2.1.1) and HISAT2 (v2.1.0) software. Gene transcript levels were estimated with RSEM (v1.3.1) and normalized as fragments per kilobase per million mapped reads (FPKM) [38]. Differentially expressed genes (DEGs) were identified using the DESeq R program (v1.24.0). Genes with a false discovery rate (FDR) < 0.05 and |log_2_ (fold change)| ≥ 1 were considered significantly differentially expressed. Functional annotation was conducted using Diamond (v0.9.24), and KEGG enrichment analysis (*P* value <.05) was carried out on the Majorbio cloud platform.

To confirm the RNA-seq results, four JA pathway-related genes that were significantly upregulated in the SC-inoculated vs non-inoculated group were selected for reverse transcription quantitative real-time polymerase chain reaction (RT-qPCR). Gene-specific primers were designed using Primer3 Plus (Table S1) and synthesized by Tsingke. Reactions were performed in 10 μL volumes using Hieff qPCR SYBR Green Master Mix (Yeasen, China) on a Quant Studio 6 Flex system (Applied Biosystems, USA), following the manufacturer’s manuals. *PpTEF2* (*TRANSLATION ELONGATION FACTOR 2*) was used as endogenous control to normalize gene transcript abundance, which was quantified using the 2^−ΔΔCT^ method with three biological and three technical replicates per sample [29].

### Physio-biochemical assays

Chlorophyll fluorescence, chlorophyll content, root vigor, hydrogen peroxide (H_2_O_2_) content, and antioxidant enzyme activities were determined according to previously described methods [37]. The maximum quantum efficiency of photosystem II (*Fv/Fm*) was measured with an IMAGING-PAM fluorimeter (Walz, Effeltrich, Germany) after 30 min of dark adaptation using the Imaging WinGegE software. Chlorophyll content was quantified by extracting 0.1 g of fresh leaf tissue in 10 ml of 80% acetone. Absorbance was measured at 663 and 645 nm using a spectrophotometer (Shimadzu, Japan), with 80% acetone as the blank. Root vigor was assessed using the triphenyl tetrazolium chloride method and expressed as deoxidization capacity (μg g^−1^ FW h^−1^). H_2_O_2_ and the activities of superoxide dismutase (SOD) and catalase (CAT) were quantified with commercial kits (Geruisi Biotechnology, China; H_2_O_2_: G0112W; SOD: G0101W; CAT: G0105W). Soil urease and polyphenol oxidase activities were determined using Boxbio kits (Beijing, China; AKEN023M and AKEN002M), following the manufacturers’ protocols. Soil total nitrogen was determined using an elemental analyzer (Elementar, Germany), and ammonium nitrogen (NH₄^+^-N) was extracted with 2 M KCl and quantified using the salicylate colorimetric method with a flow injection analyzer at 660 nm. Root architecture was analyzed with the WinRHIZO image analysis system (v2019a, Canada).

### Statistical analyses

Statistical analyses were performed using Excel 2021, SPSS v22, and GraphPad Prism v9.0. Statistical significance of differences among multiple groups was determined using one-way analysis of variance followed by Duncan’s multiple range test. Comparisons between two samples were performed using two-tailed Student’s *t* test.

## Results

### Soil metabolomics reveals amygdalin build-up with continuous peach cultivation

The phenotype of the peach trees from the three investigated plots revealed symptoms of soil-borne toxicity: the trunks exhibited gummosis lesions, likely caused by an aboveground fungal disease [29], and visual observations showed a decrease in lateral roots with increasing duration of peach cultivation (Fig. 1A). Although these observations were not quantitative, the symptoms were identified as typical of replant disease, consistent with those described in other perennial crops such as apple [39]. As a first step toward understanding the underlying causes of this phenomenon and elucidating potential changes in root rhizodeposition, non-targeted (liquid chromatography–mass spectrometry) analysis was conducted to characterize the soil metabolome across different plots.

**Figure 2:**
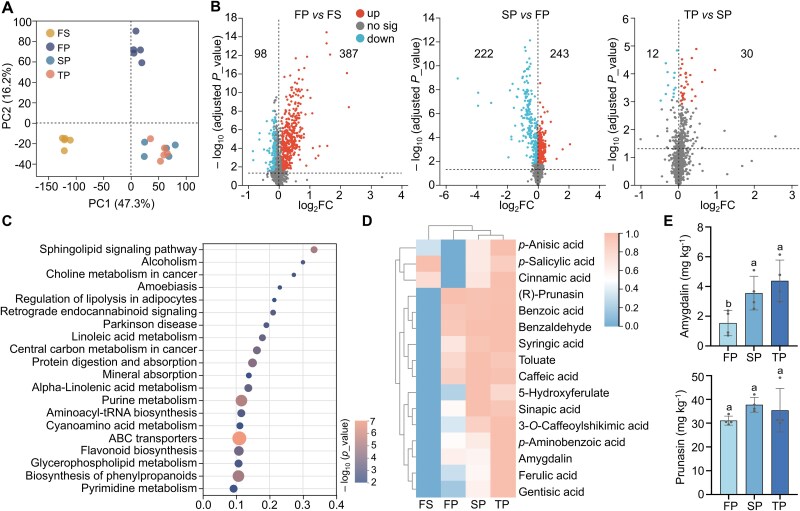
Changes in metabolite profiles across fallow soil (FS) and rhizosphere soils from the first (FP), second (SP), and third (TP) planting cycles. (A) Principal component analysis of soil metabolomic profiles. Group differences were assessed by PERMANOVA (adonis) on a Euclidean distance matrix derived from the metabolomic data matrix (*R*^2^ = 0.85, *P* value = .001). (B) Volcano plot of differential metabolites, with log_2_ fold change (FC) on the x-axis and − log_10_ (adjusted *P* value) on the y-axis. *P* values were adjusted using the FDR method. Metabolites with FDR < 0.05 and variable importance in projection (VIP) > 1 were considered significantly differential. (C) KEGG pathway enrichment of identified metabolites in all samples visualized as a bubble plot. Bubble size represents the number of metabolites, and bubble color indicates increasing significance (smaller *P* value). (D) Heatmap with hierarchical clustering of representative benzoic acid derivatives, hydroxycinnamic acid derivatives, and cyanogenic glycosides in soils. A dendrogram was generated to show sample differences and is indicated on the left side of the heatmap. Sample similarities were calculated using Bray–Curtis dissimilarities. (E) The contents of cyanogenic glycosides, amygdalin, and prunasin, across different replanting cycles. Data represent means ± SD of four biological replicates. Different letters on top of bars indicate statistically significant differences (*P* < .05). Samples for this analysis were obtained from the trial pictured in Fig. 1A.

A total of 1393 metabolites were detected. The predominant categories included lipids and lipid-like molecules (46%), organic oxygen compounds (12%), organoheterocyclic compounds (12%), organic acids and derivatives (10%), phenylpropanoids and polyketides (9%), benzenoids (6%; Fig. S1). Principal component analysis (PCA) revealed a clear separation of samples based on planting history. The first principal component (PC1) was mainly determined by the presence of peach trees, separating soils with and without peach cultivation along the x-axis, whereas the second principal component (PC2) clearly distinguished between the first planting and the replanted groups (second and third plantings) along the y-axis, indicating that continuous peach cultivation significantly reshaped the rhizosphere metabolite composition over time (Adonis: *R*^2^ = 0.85, *P* value = .001, Fig. 2A). Volcano plot analysis revealed 243 metabolites significantly upregulated and 222 downregulated in the SP group compared to the FP, whereas 30 were upregulated and 12 downregulated in the TP relative to the SP (Fig. 2B).

KEGG pathway enrichment analysis of all samples identified several key metabolic pathways and processes affected by peach cultivation, including pyrimidine metabolism, phenylpropanoid biosynthesis, glycerophospholipid metabolism, flavonoid biosynthesis, ABC transporters, and cyanoamino acid metabolism. The results suggest that over time, root-associated metabolic processes—particularly in secondary metabolism, membrane structure maintenance, and metabolite transport—and rhizodeposition lead to the build-up of metabolites in the soil of peach orchards (Fig. 2C). Sixteen differentially accumulated metabolites, such as benzoic acid, cinnamic acids, cyanogenic glycosides, and their derivatives, were significantly enriched in the replanted groups compared to the FP (Fig. 2D; amygdalin and prunasin were not detected in FS samples). Targeted HPLC analysis further confirmed that the concentrations of cyanogenic glycosides, amygdalin and its precursor prunasin, were more elevated in the rhizosphere of SP and TP fields compared to the FP, with significant values for amygdalin (Fig. 2E). These trends accurately reflect the ones observed in the non-targeted analysis (Fig. 2D).

### Amygdalin treatment compromises peach seedling growth, and soil microbes mitigate the effect

**Figure 3:**
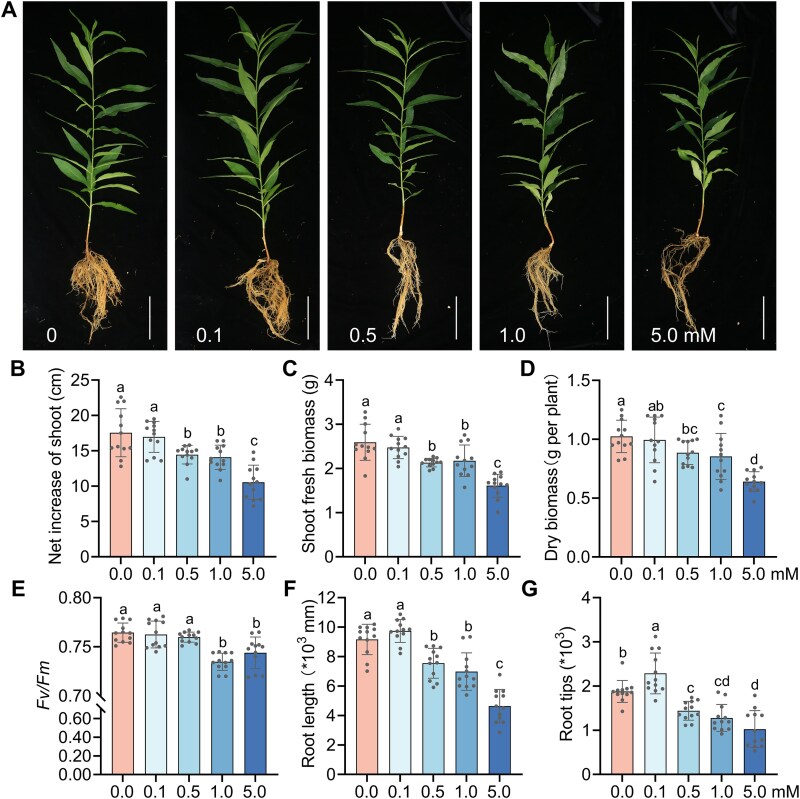
Plant performance in response to amygdalin treatments in non-sterile fallow soil. (A) Phenotypes of peach seedlings exposed to different amygdalin concentrations (0, 0.1, 0.5, 1.0, and 5.0 mM). Scale bar = 5 cm. (B–G) Effects of amygdalin on seedlings growth and physiological traits: net shoot elongation (B), shoot fresh weight (C), plant dry biomass (D), maximum PSII efficiency *Fv/Fm* (E), root length (F), and number of root tips (G). Data represent means ± SD, *n* = 12. Different letters on top of bars indicate statistically significant differences (*P* < .05). See Fig. 1B for the schematic of the experimental workflow.

To assess the autotoxic effects of amygdalin, a concentration gradient was applied over 25 d on peach seedlings grown in FS (Fig. 1B). Amygdalin exposure induced dose-dependent phenotypic changes, including lower plant height and biomass, reduced photosynthetic capacity (*Fv/Fm*) and altered root morphology (Fig. 3A–E; Fig. S2A). The root systems exhibited particular sensitivity: at 22.85, 45.70, and 228.50 mg kg^−1^ amygdalin, root length was reduced by 18%, 24%, and 49%, and root tip numbers decreased by 23%, 32%, and 45%, respectively, compared to the control (Fig. 3F and G; Fig. S2B).

**Figure 4:**
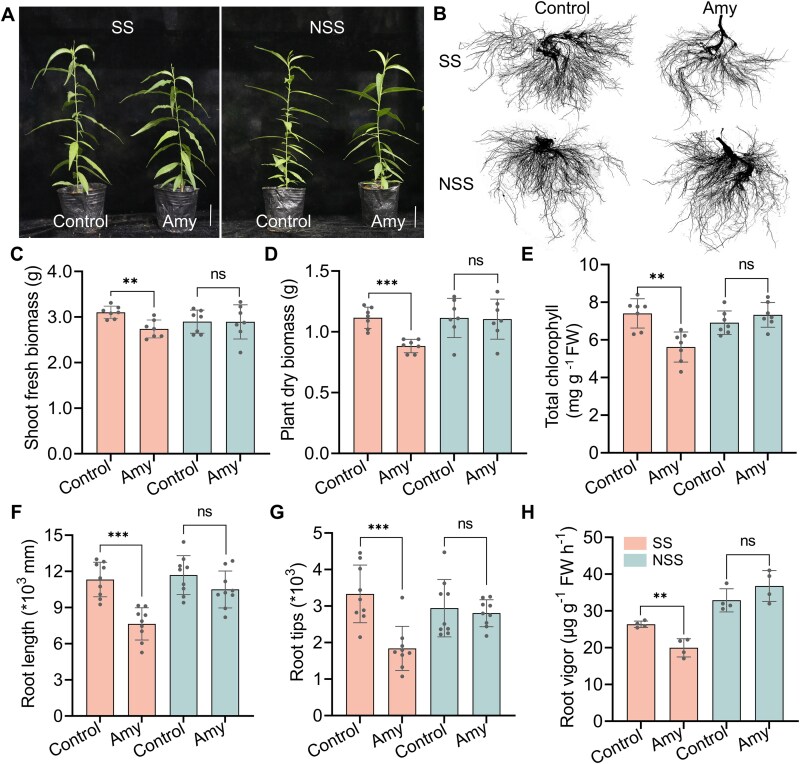
Effects of amygdalin on peach seedling growth under sterilized (SS) and non-sterilized (NSS) soil conditions. (A) Representative image of seedlings grown in SS and NSS with 0.1 mM amygdalin (Amy) or no treatment (Control). Scale bar = 5 cm. (B) Morphological changes of the root systems. (C–H) Quantitative analysis of growth parameters: Shoot fresh weight (C, *n* = 7), total plant dry weight (D, *n* = 7), total chlorophyll (E, *n* = 7), total root length (F, *n* = 9), number of root tips (G, *n* = 9), and root vigor assessed by the triphenyl tetrazolium chloride method (H, *n* = 4**)**. Data represent means ± SD. Statistical significances were calculated using a two-tailed Student’s *t*-test (ns: not significant, and ^**^P < .01; ^***^P < .001). See Fig. 1C for the schematic of the experimental workflow.

To investigate whether soil microbes modulate amygdalin toxicity on plants, seedlings were grown for 40 d in sterilized or non-sterilized FS supplemented with 0.1 mM amygdalin (4.57 mg kg^−1^ in the soil)—a concentration previously shown to lack growth-inhibitory effects and even to enhance root tip numbers (Fig. 1C; Fig. 3G). In non-sterilized soil-potted plants, amygdalin exposure did not affect root length and even enhanced mean total chlorophyll content and root vigor by 6% and 12%, respectively, with no significant inhibition of overall plant growth (Fig. 4), confirming the previous findings (Fig. 3). In contrast, seedlings in sterilized soil exhibited significant reductions in shoot fresh biomass (−12%), total dry biomass (−21%), chlorophyll content (−24%), root length (−32%), root tips (−45%), and root vigor (−24%) when treated with 0.1 mM amygdalin (Fig. 4C–H). Together, these results demonstrate that native soil microbiota buffer the phytotoxic effects of low-dose amygdalin, thereby helping sustain plant growth under autotoxic stress.

### Amygdalin recruits specific bacterial taxa in the rhizosphere

**Figure 5:**
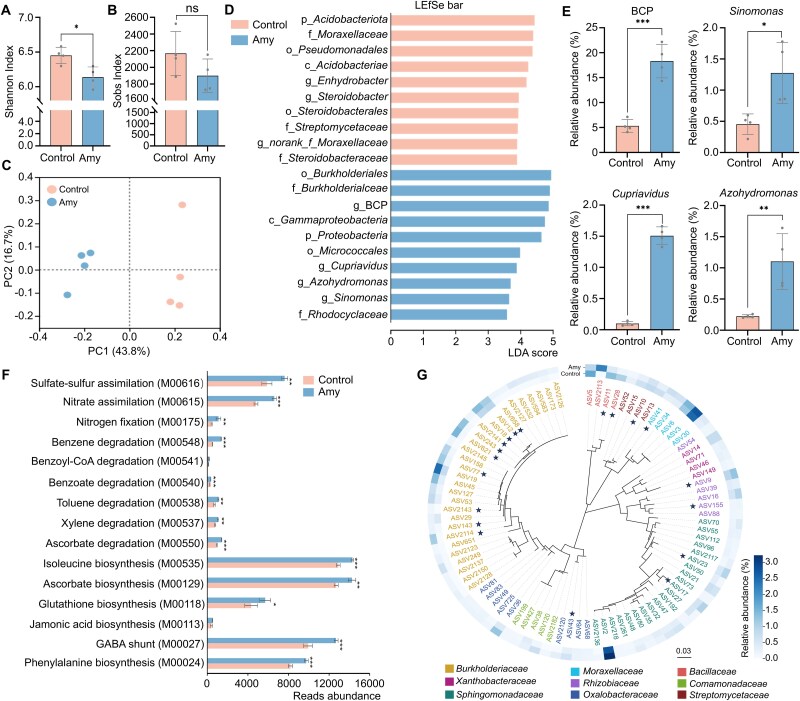
Shifts in peach rhizosphere microbial communities in response to exogenous amygdalin. (A and B) Differences in α-diversity metrics, Shannon index and Sobs index, between rhizosphere microbial communities under control (water) and 0.1 mM amygdalin treatment. (C) Principal coordinates analysis of community composition based on Bray–Curtis dissimilarities (Adonis: *P* = .027). (D) LEfSe analysis showing major taxonomic biomarkers after exogenous amygdalin treatment. LDA scores represent the contribution of differential lineages, and lineages with LDA scores >3.5 are displayed. BCP: *Burkholderia-Caballeronia-Paraburkholderia* genus. (E) Differentially enriched marker genera in rhizosphere soils. (F) Functional predictions using PICRUSt2, highlighting pathways related to stress resistance, nutrient cycling, and catabolic pathways for the degradation of benzene derivatives. (G) Phylogenetic tree of core ASVs (relative abundance >0.1%, prevalence = 1) in amygdalin-treated rhizosphere. Heatmap shows the relative abundance of core ASVs. Strains with >97% sequence similarity to these ASVs are indicated with asterisks. Scale bar = 0.03. Data represent means ± SD of four biological replicates. Statistical significances were calculated using a two-sided Student’s *t*-test (ns: not significant, and ^*^P < .05, ^**^P < .01, ^***^P < .001). Samples used for this analysis were obtained from the experimental design shown in Fig. 1C.

To assess the impact of amygdalin on microbial community assembly in the rhizosphere, we performed 16S rRNA gene amplicon sequencing on rhizosphere soil samples collected from peach seedlings grown in non-sterilized FS in pot experiment 2 (Fig. 1C). After DADA2-based processing, each sample retained 47 217 high-quality sequences. In total, 16 266 ASVs were identified across all samples. Alpha diversity analysis showed a reduction in microbial complexity under amygdalin treatment, with a significant decrease in the Shannon Index and a declining trend in the Sobs Index (Fig. 5A and B). Principal coordinates analysis based on Bray–Curtis dissimilarities revealed a clear separation between amygdalin-treated (Amy) and water (Control) rhizosphere communities (Adonis, *P* value = .027), confirming structural shifts in response to amygdalin addition (Fig. 5C). Based on the ASV classification, the dominant phyla across all samples were *Proteobacteria*, *Actinobacteria*, *Acidobacteria*, and *Chloroflexi*, collectively accounting for 77%–87% of the total reads (Fig. S3A and B). To pinpoint biomarker rhizobacterial taxa associated with community differentiation, LEfSe analysis identified several bacterial genera that were significantly enriched in amygdalin-treated soils, including *Burkholderia-Caballeronia-Paraburkholderia*, *Cupriavidus*, *Azohydromonas*, and *Sinomonas* (LDA score > 3.5, *P* value <.05; Fig. 5D and E; Fig. S3C). The *Burkholderia-Caballeronia-Paraburkholderia* group exhibited a 243% increase in relative abundance compared to the control, emerging as the most abundant taxon in the rhizosphere of amygdalin-treated plants (Fig. 5E; Fig. S3C).

PICRUSt2 was used to predict KEGG MODULE abundances related to nutrient cycling, and stress resilience from 16S rRNA gene data. Compared with the control, amygdalin-treated samples showed significantly higher predicted abundances in seven MODULEs involved in antioxidant and defense compound biosynthesis (M00024, M00027, M00113, M00118, M00129, M00535, M00550), four MODULEs associated with aromatic compound degradation (M00537, M00538, M00540, M00548), and three MODULEs related to nutrient cycling (M00175, M00615, M00616; Fig. 5F). PICRUSt2 predictions suggested that amygdalin drives compositional restructuring of the rhizosphere microbial community, selecting taxa with projected enhanced capacities in antioxidant and defense compounds biosynthesis, aromatic compound degradation, and nutrient cycling.

### Tolerance and degradation of amygdalin by rhizobacteria may drive their enrichment in the peach rhizosphere

We isolated and purified 104 bacterial strains from the rhizosphere soil of peach seedlings grown in non-sterilized FS in pot experiment 2 (Fig. 1C). From these, representative isolates were selected based on 97% sequence identity between their 16S rRNA gene amplicons and ASVs in amygdalin-treated soils, applying the following criteria: prevalence = 1, total relative abundance >0.1% of all samples under amygdalin treatment, and representation by more than one ASV per genus. This selection process yielded 19 representative strains covering all major families except for *Xanthobacteraceae* and *Comamonadaceae* (Fig. 5G; Table S2).

**Figure 6:**
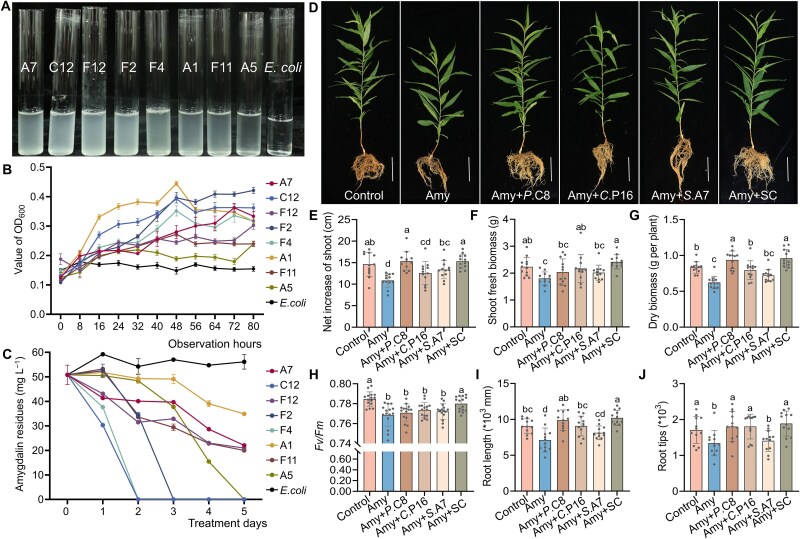
Growth-promoting effects of three bacterial strains (alone or in combination) on peach seedling growth under 1 mM amygdalin treatment. (A) Culture turbidity of eight rhizosphere bacteria isolated from the rhizosphere of peach seedlings grown in amygdalin-treated FS (A7, C12, F12, F2, F4, A1, F11, and A5) and *Escherichia coli* DH5α (control) during 5 d of culturing in MSM supplemented with amygdalin. A7: *Sinomonas humi*; C12: *Burkholderia* sp.; F12: *Paraburkholderia* sp*.*; F2: *Paraburkholderia* sp*.*; F4: *Burkholderia* sp*.*; A1: *Paraburkholderia* sp*.*; F11: *Burkholderia* sp*.*; A5: *Novosphingobium* sp. (B) Growth curves of the isolates in amygdalin-containing medium over 80 h, monitored by OD_600_ at 8 h intervals. Data are mean ± SE, *n* = 7. (C) Degradation efficiency was analyzed by measuring residual amygdalin (from 50 mg l^−1^ initial concentration) in MSM after 5 d of incubation with each isolate, with *E. coli* (unable to degrade amygdalin) as a negative control. Data are means ± SD, *n* = 3. The schematic workflow is shown in Fig. 1D. (D) Phenotypes of peach seedlings inoculated with three single strains (*Priestia megaterium* C8, *Caballeronia turbans* P16 and *S. humi* A7) and their combination (SC), or not inoculated under amygdalin treatment (Amy), plus uninoculated and untreated seedlings (Control). Scale bar = 5 cm. (E–J) Growth and physiological performance of peach seedlings inoculated with different strains: net shoot elongation (E), shoot fresh weight (F), plant dry biomass (G), maximum PSII efficiency *Fv/Fm* (H), root length (I), and number of root tips (J). Data represent means ± SD, *n* = 12. Different letters on top of bars indicate significant differences among treatments (*P* < .05). The experimental workflow is shown in Fig. 1F.

Given that amygdalin itself and its degradation products, including hydrogen cyanide, benzaldehyde-type aromatics, cyanogenic derivatives, and glycosidic forms, are inhibitors of bacterial growth [40, 41]. To assess potential rhizobacterial tolerance, we compared the growth of the 19 selected rhizobacterial strains and *Escherichia coli* DH5α under exposure to varying concentrations of amygdalin and of its soil-metabolized derivatives collected at different time points. Growth was monitored by OD_600_ every 8 h over 56 h. Whereas *E. coli* DH5α showed high susceptibility to amygdalin, the majority of rhizobacterial isolates exhibited normal growth. Two strains, A7 and A2, demonstrated significantly enhanced growth after 24 h in the presence of amygdalin (Fig. S4). Similarly, soil-metabolized amygdalin derivatives strongly inhibited *E. coli* DH5α but promoted the growth of five strains (P16, A2, F11, A5, and D2), with negligible effects observed on others (Fig. S5). We further assessed the capacity of these isolates to utilize amygdalin as the sole carbon source in MSM. Strains A7 (*Sinomonas*), C12, F12, F2, F4, A1, and F11 (*Burkholderia-Caballeronia-Paraburkholderia* complex), and A5 (*Novosphingobium*) were able to not only grow under these conditions but also degrade amygdalin effectively (Fig. 6A–C). The observed features of these eight strains, which were previously identified as enriched under amygdalin exposure (Fig. 5G; Table S2), suggest that their metabolic capacity likely contributes to their ecological success in the amygdalin-contaminated rhizosphere.

### Bacterial inoculation restores growth performance in amygdalin-treated peach seedlings

Given that amygdalin directly restricts root elongation and development, we inoculated germinated seeds with isolated bacterial strains and sprayed with 0.5 mM amygdalin, then evaluated seedling growth after 7 d (Fig. 1E). Inoculation with strains C8, P16, A7, P25, A2, F2, E8, A1, F4, C12, or F11 promoted root development of peach seedlings (Fig. S6A), with root length significantly increased by 43%–147% compared with the uninoculated, amygdalin-treated control. Among these, strains C8, P16, and A7 exhibited the most pronounced effects in alleviating inhibition (Fig. S6B). These three isolates, C8 (*Priestia megaterium*), P16 (*Caballeronia turbans*), and A7 (*Sinomonas humi*), were prioritized for SC assembly because they are ecologically representative core bacterial taxa, individually showed the strongest alleviating effects, and displayed complementary functional traits related to amygdalin tolerance or metabolism (Fig. 6D–J; Figs S4–S7). A pot experiment was conducted for one month in sterilized FS under 1 mM amygdalin stress to evaluate the effects of single-strain inoculation and of their co-inoculation on peach seedling growth (Fig. 1F). Amygdalin treatment significantly inhibited seedling growth compared with the control, as evidenced by decreases of 25% in plant dry weight and 22% in root length, whereas inoculation with any of these strains mitigated such inhibition, enhancing shoot, root, and total plant length, and improving overall growth indices (Fig. 6D–J). The SC inoculation conferred the most robust restorative effect, outperforming not only the amygdalin-treated control but also the seedlings treated with individual strains. The SC-inoculated seedlings exhibited expanded root architecture, with root length and root tip number increasing by 43% and 41% compared to uninoculated, amygdalin-treated seedlings, respectively. They outperformed even the mock-treated controls that were not exposed to amygdalin, compared to which root length and root tip number were increased by 13% and 11%, respectively (Fig. 6I and J). Also in non-sterile soil, SC inoculation mitigated amygdalin-induced stress, resulting in a 40% increase in plant dry weight (Fig. S8), although this increase was lower than that observed in sterile soil (54%, Fig. 6D). These findings indicate that the selected strains, particularly if inoculated as a SC, can effectively alleviate amygdalin-induced phytotoxicity and restore normal growth in peach seedlings, even in a competitive environment.

### Synthetic community-induced plant resistance against amygdalin stress

**Figure 7:**
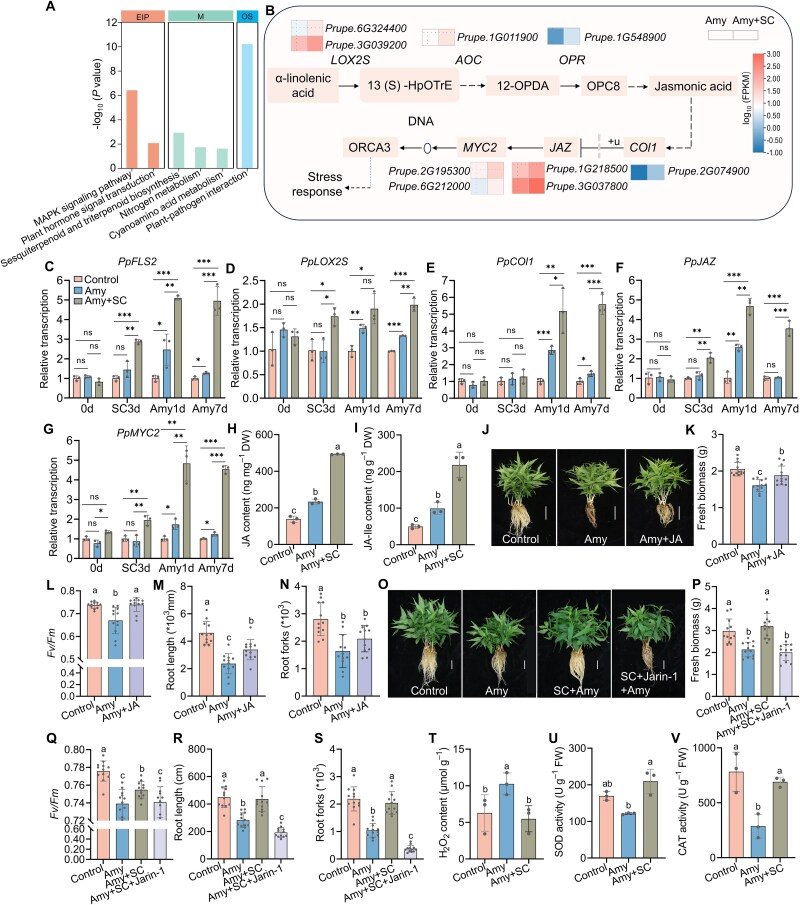
Biological mechanisms underlying SC-mediated enhancement of peach seedling tolerance to amygdalin-induced inhibition. (A) KEGG pathway enrichment revealing functional shifts in the roots of SC-inoculated seedlings under amygdalin treatment (Amy vs Amy + SC). EIP: Environmental Information Processing; M: Metabolism; OS: Organismal Systems. (B) JA biosynthesis and signaling pathway with heatmaps showing log_10_ (FPKM) of DEGs. (C–G) Relative transcript abundance of the pattern recognition receptor gene *PpFLS2* (*FLAGELLIN SENSING 2*), the JA biosynthesis gene *PpLOX2S* (*LINOLEATE 13S-LIPOXYGENASE*), and JA signaling genes *PpCOI1* (*CORONATINE-INSENSITIVE 1*)*, PpJAZ* (*JASMONATE ZIM-DOMAINS*)*, and PpMYC2* (*MYELOCYTOMATOSIS ONCOGENE HOMOLOG 2*) in response to amygdalin treatment and/or SC inoculation as in (A)*.* Gene transcripts were normalized to the values of *PpTEF2* (*TRANSLATIONAL ELONGATION FACTOR 2*), and the untreated control samples. 0 d: untreated; SC3d: 3 d of SC pre-inoculation before amygdalin treatment. Amy1d and Amy7d: 1 and 7 d after amygdalin addition following 3 d of SC pre-inoculation. Data represent means ± SD of three biological replicates. (H and I) Quantification of JA and JA-Ile contents in amygdalin-treated and/or SC-inoculated roots (7 d) roots after 7 d, compared with untreated and uninoculated roots. Data represent means ± SD of three biological replicates. (J) Phenotypes of hydroponically grown peach seedlings under control conditions, 0.5 mM amygdalin (Amy), and 0.5 mM amygdalin following pretreatment with 100 μM JA (Amy + JA), *n* = 9. Scale bar = 5 cm. (K–N) Quantitative analysis of the alleviatory effect of 100 μM exogenous JA on amygdalin-induced growth inhibition, *n* = 12. (O) Phenotypes of peach seedlings grown hydroponically for 10 d under control conditions, 0.5 mM amygdalin (Amy), SC inoculation for 2 h followed by 3 d of cultivation in Hoagland solution before 0.5 mM amygdalin treatment (Amy + SC), and SC inoculation for 2 h followed by 3 d of cultivation in Hoagland solution, 2 h of pretreatment with 10 μM Jarin-1, and subsequent 0.5 mM amygdalin treatment (Amy + SC + Jarin-1), *n* = 12. Scale bar = 5 cm. (P–S) Morphological quantification of Jarin-1 effects on the SC-mediated amygdalin stress alleviation (*n* = 12). (T–V) H_2_O_2_ content and activities of CAT and SOD in SC-inoculated roots under amygdalin treatment. Data represent means ± SD of three biological replicates. Asterisks indicate statistical significance using a two-sided Student’s *t*-test (ns: not significant, ^*^P ≤ .05, ^**^P ≤ .01, and ^***^P ≤ .001). Different letters on top of bars indicate significant differences between different treatments (*P* < .05). The experimental scheme is shown in Fig. 1F, G, and  H.

To elucidate the mechanisms underlying the beneficial effects of SC inoculation on plant performance in amygdalin-treated soil, we compared root transcriptomes of peach seedlings subjected to amygdalin stress for 7 d, with or without SC inoculation. Under amygdalin treatment (1 mM), inoculation resulted in 778 DEGs (621 upregulated and 157 downregulated) compared to non-inoculated controls (Table S3). These DEGs were enriched in several key KEGG pathways related to plant defense and immune responses, such as plant–pathogen interaction, MAPK signaling pathway, and plant hormone signal transduction (Fig. 7A; Fig. S9). Nine genes associated with the JA biosynthesis and signaling pathway were upregulated. These include two putative *LINOLEATE 13S-LIPOXYGENASES* (*LOX2S*s), one *ALLENE OXIDE CYCLASE* (*AOC*), and one *12-OXOPHYTODIENOATE REDUCTASE* (*OPR*), all of which are involved in JA biosynthesis. We also identified JA signaling components, including one *CORONATINE-INSENSITIVE 1* (*COI1*), two *JASMONATE ZIM-DOMAINS* (*JAZ*s), and two genes encoding MYELOCYTOMATOSIS ONCOGENE HOMOLOG 2 (MYC2). COI1 encodes the receptor and activator of JA signaling, JAZ encodes repressor of JA-responsive gene transcription, and *MYC2* encodes a central transcription factor in JA signaling (Fig. 7B).

To further verify the modulation of microbial perception components and of the JA pathway in roots exposed to amygdalin and the SC, we performed RT-qPCR analysis of *FLS2* (*FLAGELLIN SENSING 2*, which encodes the receptor for the bacterial elicitor flagellin) and representative JA-related genes across different treatment stages, including pre-inoculation, 3 d after SC inoculation, as well as 1 and 7 d after amygdalin treatment in the presence or absence of SC inoculation (Fig. 7C). In comparison with uninoculated roots, SC inoculation significantly increased the transcription of *FLS2* regardless of amygdalin treatment. Furthermore, the SC consistently and significantly enhanced the transcription of JA pathway marker genes (*LOX2S*, *COI1, JAZ,* and *MYC2*) in amygdalin-treated roots compared to the uninoculated under amygdalin stress (Fig. 7D–G).

Consistent with a mechanism of action mediated by transcriptional activation of JA biosynthesis, SC inoculation also significantly increased the contents of JA and JA-Ile in roots compared to both the uninoculated, amygdalin-treated samples and especially the uninoculated and untreated controls (Fig. 7H and I). This suggests that the presence of the SC enhances a JA-mediated tolerance mechanism which is not as efficiently triggered by amygdalin itself. To test this hypothesis, the roots of peach seedlings were dipped in 100 μM JA for 12 h, and then cultured in Hoagland nutrient solution containing 0.5 mM amygdalin for 7 d (experimental setup in Fig. 1G). JA application significantly mitigated the inhibitory effects of amygdalin on plant parameters, as evidenced by increases in plant biomass, photosynthetic capacity, and root length in amygdalin-treated plants exposed to JA vs the unexposed (Fig. 7J–N). In contrast, application of 10 μM Jarin-1 (Fig. 1H), a JA-Ile biosynthesis and signaling inhibitor previously reported to suppress over 70% of JA responses in Arabidopsis at this concentration, reduced the expression of JA biosynthesis- and signaling-related genes, but having no significant effect on *FLS2* expression (Fig. S10). Jarin-1 treatment significantly attenuated the SC-mediated alleviation of amygdalin stress in peach seedlings (Fig. 7O–S), further supporting a central role for the JA pathway in SC-mediated amygdalin tolerance.

Compared with the water-treated control, amygdalin treatment resulted in a substantial accumulation of H_2_O_2_ in peach roots and suppressed the activities of the key antioxidant enzymes SOD and especially CAT. In contrast, SC inoculation significantly alleviated the amygdalin-induced suppression of both SOD and CAT activities, thereby promoting more efficient scavenging of reactive oxygen species (ROS) such as H_2_O_2_ (Fig. 7T–V). Together, our results suggest that SC inoculation enhances peach seedling tolerance to amygdalin by elevating JA biosynthesis and thus, downstream signaling, and maintaining ROS homeostasis by protecting the antioxidant system.

### The synthetic community colonizes peach roots and modifies rhizosphere traits

**Figure 8:**
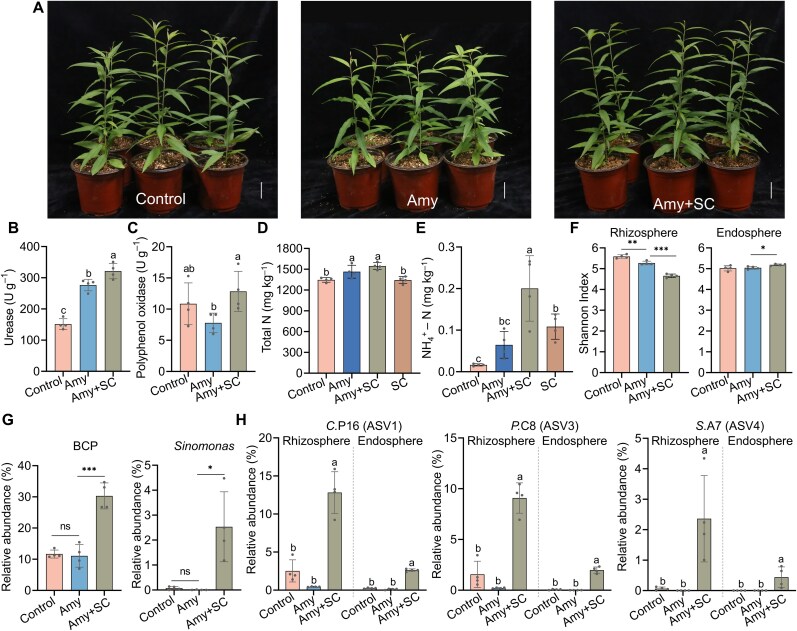
Effects of microbial inoculation on soil enzyme activities and rhizosphere/root microbiota of peach plants grown in sterilized FS under amygdalin-induced stress. (A) Growth performance of peach seedlings two months after SC inoculation under 1 mM amygdalin treatment. Scale bar = 5 cm. (B and C) Activities of soil urease and polyphenol oxidase (One unit (U) of polyphenol oxidase activity was defined as the amount of enzyme that produces 1 mg of purpurogallin per gram of soil per day) in peach rhizosphere as in (A). (D and E) Soil contents of total nitrogen and ammonium nitrogen (NH_4_^+^-N). SC: inoculated with SC only without amygdalin treatment. (F) Changes in the Shannon index in peach rhizosphere and roots as in (A). (G) Relative abundances of *Burkholderia-Caballeronia-Paraburkholderia* (BCP) and *Sinomonas* in the rhizosphere of peach seedlings. (H) Relative abundances of the bacterial ASVs in the rhizosphere and endosphere of 2-month-old peach plants with or without SC inoculation under amygdalin treatment as in panel (A). The three ASVs showing a perfect match to the V5–V7 region of the isolated strains are highlighted. Data represent means ± SD of four biological replicates. Asterisks indicate statistical significance using a two-sided Student’s *t*-test (^*^P ≤ .05, ^**^P ≤ .01, and ^***^P ≤ .001). Different letters on top of bars indicate statistical significances between different treatments (*P* < .05). Figure 1F shows the experimental scheme.

To investigate the effects of SC inoculation on soil nutrient cycling and microbial community composition, peach seedlings were grown in sterilized FS inoculated, or not, with the SC. After 2 months of amygdalin treatment applied at 1 mM every 5 d, rhizosphere soil samples were collected for the analysis of enzyme activities and microbial community composition. Because amygdalin toxicity is largely attributed to its benzene ring and cyanide group, we measured the activities of polyphenol oxidase (which catalyzes the oxidation of aromatic compounds) and urease (which facilitates nitrogen cycling) [42, 43]. Results confirmed that amygdalin suppressed plant growth after 2 months; however, inoculation noticeably alleviated this inhibitory effect (Fig. 8A) consistently with the results of pilot experiments (Fig. 6D–J). For soil enzymes, amygdalin addition alone elevated soil urease activity by 112% compared to the untreated control. Under amygdalin stress, inoculation significantly enhanced the activities of polyphenol oxidase and urease by 65% and 16%, respectively, relative to the non-inoculated group (Fig. 8B and C). Total nitrogen content was12% higher in the amygdalin-treated soils (Amy and Amy + SC) than in the uninoculated and untreated control, whereas NH_4_^+^-N content increased 12.1-fold in the Amy + SC group in relation to the control samples (Fig. 8D and E). This implies that SC inoculation accelerates the degradation of allelochemicals and their conversion into plant-available nutrients.

Root colonization extent under specific treatments is widely used in microbiome studies as a key indicator of the ecological relevance of specific bacterial strains [44]. To evaluate root colonization by the SC and its impact on the resident microbiota, we performed amplicon sequencing of rhizosphere and root endosphere samples. Under amygdalin stress, inoculation significantly reduced the Shannon Index of the rhizosphere microbial community but increased diversity in the root compartment (Fig. 8F). Furthermore, amygdalin treatment did not enrich *Burkholderia-Caballeronia-Paraburkholderia* and *Sinomonas* in sterilized soil, in contrast to non-sterilized soil conditions (Figs 5E and 8G). Using the V5–V7 region of the 16S rRNA gene as a taxonomic marker, we matched the three inoculated strains to specific ASVs (Table S4). The three SC strains, ASV1 (*C. turbans* P16), ASV3 (*P. megaterium* C8), and ASV4 (*S. humi* A7), exhibited significantly higher relative abundances in both the rhizosphere and root endosphere compared to uninoculated controls (Fig. 8H), suggesting the successful root colonization by SC constituents.

## Discussion

By integrating field surveys, multi-omics analyses, pot and hydroponic experiments, we identified amygdalin as a representative allelopathic autotoxin that accumulates in the rhizosphere of peach trees with increasing duration of continuous cultivation. Furthermore, we revealed that amygdalin drives the assembly of a protective rhizosphere microbiome. Based on this, a simplified SC comprising three strains, through amygdalin detoxification and modulation of the JA pathway and ROS homeostasis in the plants, contributes to maintaining plant health.

### Allelochemical accumulation under replanting systems contributes to negative soil–plant feedback

Perennial plants, characterized by extended growth cycles and persistent root–soil interactions, are particularly prone to the accumulation of allelopathic substances in the rhizosphere [45–47]. Although these compounds play roles in plant defense under natural conditions, their persistent buildup under continuous monocropping can disrupt soil ecology and plant performance, causing or exacerbating replant problems [48]. For instance, in apple replant systems, phlorizin and benzoic acid have been identified as major allelopathic compounds that inhibit root cell division and suppress lateral root formation, ultimately leading to replant disease [49]. Similarly, in *Panax notoginseng* and *Camellia sinensis*, the long-term accumulation of phenolic acids has been associated with rhizosphere microbial dysbiosis and severe replant failure [50, 51]. Our results confirmed that such metabolite accumulation also occurs in replanted peach orchards. Metabolomic profiling revealed significant build-up of cyanogenic glycosides and phenolic acids in the replanted peach rhizosphere. This pattern is characteristic of negative soil–plant feedback, indicating that plant-derived metabolites can shift from protective agents to stressors that inhibit plant growth in replanted systems.

Cyanogenic glycosides are widely present in plant families, including legumes, *Lemnaceae*, and *Rosaceae*, where they function as defensive compounds [52, 53]. Amygdalin occurs at relatively high concentrations in peach roots (~50 mg g^−1^ dry weight) [54]. In replanted peach and apple systems, amygdalin accumulation has been associated with direct growth inhibition of tree crops [55, 56]. Microbial degradation of amygdalin produces phytotoxic intermediates such as benzoic acid, benzaldehyde, and hydrogen cyanide [25, 57], raising the possibility that microbial transformation may either mitigate or exacerbate plant stress. This functional ambiguity, together with its high abundance in peach roots and sharp accumulation in peach orchard soil, provides a rationale for selecting amygdalin as a model allelochemical to investigate microbiome-mediated plant–soil interactions. When we applied exogenous amygdalin under controlled conditions, we indeed observed concentration-dependent effects on root development.

At a low concentration, amygdalin transiently stimulated root growth, consistent with previous studies that certain allelochemicals can exhibit hormetic or hormone-like properties that modulate root development, and microbial recruitment supporting plant development [16, 58], but by Day 40 this promotive effect had disappeared and root length was slightly inhibited. In FS, amygdalin became undetectable in the rhizosphere within 5 d even at 5 mM, whereas in replanted peach rhizosphere soil it remained detectable at ~0.01 mM. This contrast suggests that continuous monoculture may impair the rhizosphere’s self-remediation capacity, allowing low but persistent levels of amygdalin (and its degradation products) to accumulate and contribute to replant problems. In sterilized soil, 0.1 mM amygdalin inhibited peach seedling growth, whereas no significant inhibition occurred in non-sterile soil, suggesting that soil microbial communities contribute to the attenuation of amygdalin toxicity.

Beyond direct phytotoxicity, allelochemicals also reshape the structure of the microbial community. Compounds such as benzoic acid, hydrogen cyanide, benzaldehyde, and other phenolic derivatives exert strong selective pressure on soil microbes, often reducing overall diversity and enriching taxa with specialized functions [59]. These changes may weaken the multifunctionality of soil microbes and disrupt the functional redundancy within microbial communities. This biphasic response aligns with findings across multiple allelochemical systems, indicating that soil self-repair capacity is limited; therefore, in modern continuous-cropping systems, progressive soil degradation together with allelochemical inputs from plant residues and root exudates is likely to intensify negative soil–plant feedback [60]. Thus, the ability of allelochemicals and microbiome shifts to either relieve or contribute to toxicity under continuous monoculture on the other, likely reflect different stages and conditions of the plant–soil system.

### Amygdalin-driven recruitment of a specialized microbiome enhances plant–soil system resilience

A growing body of evidence indicates that various plant allelochemicals exhibit selective antimicrobial properties that significantly influence the composition of root-associated microbiomes [37]. Microbial community structure is thus shaped not only by competition for resources but also by varying tolerances to plant-derived biochemicals. For example, in maize roots, bacterial abundance patterns correlate with benzoxazinoid tolerance, where resistant taxa dominate and sensitive strains are suppressed [61]. This aligns with the established two-step selection model for rhizosphere assembly, wherein root exudates first induce substrate-mediated shifts in microbial composition, which is then fine-tuned by plant genotypes [62–64].

Plant-derived metabolites can act as selective forces in the rhizosphere, shaping microbial community structure and function under both normal and stress conditions [12, 65]. Our study indicates that amygdalin treatment, possibly in concert with the host plant, actively reprograms the microbiome, enhancing functions related to aromatic compound degradation, antioxidant and resistance compound biosynthesis, and nutrient assimilation; in this frame, bacterial tolerance to amygdalin appears to play a critical role. Nineteen rhizobacterial strains from the rhizosphere of amygdalin-treated seedlings showed higher tolerance to amygdalin and its derivatives than *E. coli*. Taxa such as *Burkholderia-Caballeronia-Paraburkholderia* and *Sinomonas* were significantly enriched under amygdalin-treated soils. These bacteria are able to utilize it as a sole carbon source, highlighting a dual capacity to both tolerate and catabolize cyanogenic or aromatic compounds (Fig. 6B and C; Figs S4 and S5). In particular, *Paraburkholderia* is regarded as a key taxon initiating phenolic acid degradation in soils, a function that may be critical for the detoxification of soil-borne allelochemicals [66]. Thus, amygdalin accumulation reduced overall microbial diversity but promoted functional convergence, enriching tolerant and functionally specialized lineages.

Recent studies suggest that bacterial tolerance to host-exuded specialized metabolites is often linked to structural traits such as outer membrane modifications [61], we did not observe obvious associations between amygdalin tolerance and bacterial cell envelope traits in our isolates. This discrepancy points to alternative tolerance mechanisms, such as enzymatic detoxification, efflux pumps, or transcriptional regulatory networks [67, 68]. The unraveling of these mechanisms represents a promising direction for future research. Moreover, our findings suggest that amygdalin plays a key role in recruiting functionally specialized microbes, yet this may represent only part of the mechanism; additional pathways, such as the secretion of other signaling compounds, may also contribute to rhizosphere remodeling in peach and warrant further investigation.

### Synthetic microbial community alleviates peach autotoxicity via plant jasmonic acid pathway activation

Although plants exhibit a certain degree of self-repair capacity under allelopathic stress, this intrinsic ability is insufficient to counteract the long-term consequences of inappropriate agricultural practices. Currently, the direct application of beneficial bacteria into soil is a major strategy to enhance plant growth and stress resilience; however, these introduced bacteria often fail to establish stable rhizosphere populations due to unfavorable soil environments, limited ecological niches, and competition with indigenous microorganisms. Therefore, utilizing beneficial strains derived from indigenous microbial communities may represent a more effective and sustainable solution [69]. Inoculation with toxin-degrading microbes has been widely shown to alleviate autotoxicity from allelopathic compounds by removing them and restoring soil–plant balance [23, 70–72]. In our study, *S. humi* A7 exhibited strong amygdalin-degrading ability. The SC containing *S. humi* A7 significantly enhanced soil polyphenol oxidase activity, urease activity, and ammonium content. These results suggest that inoculation with the SC facilitated the cycling and transformation of nitrogen derived from amygdalin into plant-available forms, thereby helping to offset autotoxicity and improve nutrient availability. Such functional shifts may explain why SC inoculation strongly promoted peach seedling growth under amygdalin-induced stress compared with the control. Although the degradation of amygdalin generates toxic intermediates, our findings indicate that inoculation with amygdalin-degrading bacteria remains an effective strategy to mitigate amygdalin-induced stress.

The SC inoculation also activated the JA pathway in peach roots, with strong upregulation of JA biosynthetic and signaling genes and accumulation of JA and JA-Ile in inoculated plants. Exogenous JA application partially restored growth under amygdalin stress, whereas inhibition of JA-Ile biosynthesis attenuated the alleviating effect of SC inoculation, supporting a central role for the JA pathway in mediating stress resilience. Beneficial microbes such as *Penicillium, Stenotrophomonas, Pseudomonas, Streptomyces*, and *Trichoderma* have frequently been reported to activate JA signaling to enhance plant resilience under biotic and abiotic stresses [73, 74], and JA-mediated responses have also been implicated in plant acclimation to allelopathic compounds, including to autotoxic ginsenosides in *P. ginseng* [75]. JA mitigates various chemical stresses such as salinity, heavy metals, and herbicides by activating antioxidant defenses and modulating hormone crosstalk in the producing plant, and facilitating the recruitment of beneficial microbes [76, 77]. In our system, SC inoculation did not elevate antioxidant enzyme activities beyond the control level, but significantly alleviated their suppression under amygdalin stress, accompanied by reduced H_2_O_2_ accumulation. Because cyanogenic compounds can directly inhibit the activity of ROS-scavenging enzymes and disrupt antioxidant function [78], these results suggest that SC contributes to stress alleviation mainly by maintaining redox homeostasis and protecting the antioxidant system from amygdalin-induced inhibition.

Plant hormone signaling operates as an interconnected regulatory network in which JA interacts extensively with other hormones, including ethylene (ET) and abscisic acid (ABA), to coordinate stress acclimation and growth-defense trade-offs. JA and ET are known to often act cooperatively through shared transcriptional regulators, whereas ABA signaling rather modulates stress response intensity and recovery processes [79]. Although our study primarily highlights the role of SC inoculation in alleviating amygdalin stress through the JA pathway, transcriptomic data also revealed responses in several other hormone-related pathways, including auxin (IAA), ET, ABA, brassinosteroid, gibberellin, and salicylic acid (SA) signaling (Fig. S11A). Nevertheless, targeted metabolite analysis did not detect significant increases in IAA, ABA, or SA contents following SC inoculation (Fig. S11B–D), suggesting that these pathways may not be activated through large changes in hormone accumulation under our experimental conditions. In this context, JA-mediated stress mitigation may function within a broader hormonal framework rather than as an isolated pathway. IAA-mediated root developmental responses have been proposed as an alternative mechanism by which beneficial microbes alleviate allelopathic stress [24]. However, we did not detect significant changes in root IAA levels in our system (Fig. S11B), an apparent inconsistency that may stem from differences in microbial taxa or experimental contexts.

Although our mechanistic experiments were conducted under controlled pot conditions, we further evaluated SC performance in non-sterile soil to better approximate natural environments. Although the magnitude of growth promotion was reduced relative to sterilized soil, SC inoculation still significantly mitigated amygdalin-induced stress in non-sterile FS. This suggests that the protective effect of SC is not solely an artifact of simplified soil systems but can operate under conditions of microbial competition. Nevertheless, field-level validation will be necessary to determine long-term stability and ecological persistence of the consortium. Moreover, using JA-deficient or JA-signaling mutants will be essential to definitively substantiate the involvement of the JA pathway in the observed resilience.

## Conclusions

**Figure 9:**
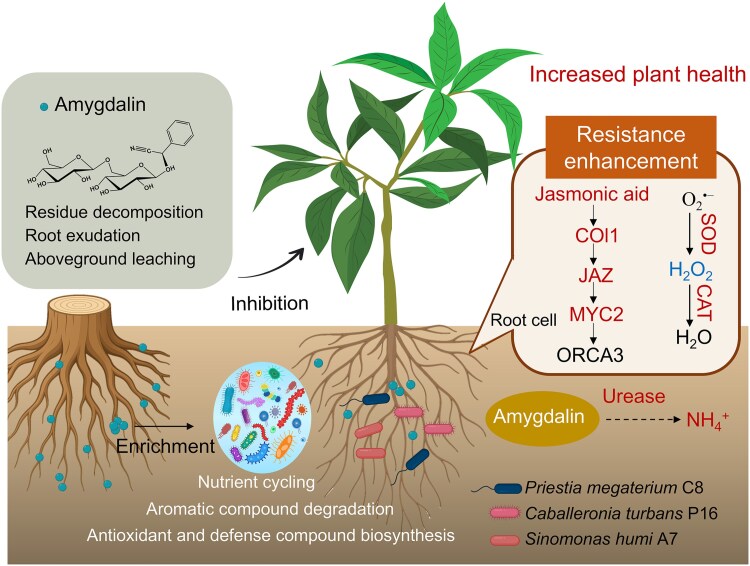
Proposed mechanisms and plant-microbiota interactions underlying the mitigation of autotoxic inhibition in peach. Amygdalin is a major plant specialized metabolite that accumulates in the rhizosphere of peach trees with successive replanting cycles, inhibiting plant growth. Application of exogenous amygdalin to peach seedlings selectively enriches distinct microbial taxa in the rhizosphere and concomitantly enhances predicted community-level functions related to nutrient cycling, aromatic compound degradation, and the biosynthesis of antioxidant and defense-related compounds. Inoculation with a simple function-specific SC assembled from rhizosphere isolates of amygdalin-treated seedlings, namely *Priestia megaterium* C8, *Caballeronia turbans* P16, and *Sinomonas humi* A7, alleviates amygdalin-induced allelopathic stress and promotes plant growth by activating jasmonate signaling and maintaining reactive oxygen species homeostasis.

This study reveals that amygdalin accumulates in the rhizosphere of long-term peach orchards, and that exogenous amygdalin inhibits seedling growth. Amygdalin reshapes the rhizosphere community, selectively pressuring for the recruitment of specific microbial taxa that increase plant tolerance. Leveraging these insights, we assembled a function-specific SC from amygdalin-treated plants, which not only facilitated amygdalin degradation but also activated the JA pathway and maintained ROS homeostasis in peach, thereby mitigating amygdalin-induced stress and improving seedling performance (Fig. 9). Our results suggest that allelopathic compounds may both cause autotoxicity and help select microbes that alleviate it, in a dynamic balance that is likely to delay the onset of severe stress. This framework also offers new avenues for microbiome-informed crop management. By designing function-specific SC and deploying it in replanted soils, it is possible to alleviate replant problems, enhance nutrient use efficiency, and stabilize root health.

## Supplementary Material

Yang_J_et_al_ms1_Suppl_figures_260408_wrag095

Yang_et_al_ms1_Supplemental_tables_260408_wrag095(1)

## Data Availability

The data in this study can be requested from the corresponding author. The raw 16S amplicon and RNA-seq data have been deposited in the Genome Sequence Archive (GSA) at the China National Center for Bioinformation, under BioProject PRJCA052010, with accessions CRA034264 and CRA034671 (16S rRNA gene amplicon sequencing), and CRA034204 (RNA-seq).

## Additional information

**Publisher’s note** Springer Nature remains neutral with regard to jurisdictional claims in published maps and institutional affiliations.
