# Supplementary material for: Rhizosphere microbial shifts drive amygdalin detoxification and jasmonate-mediated alleviation of peach autotoxicity
Source: ISME J. 2026 Apr 16;20(1):wrag095. doi: 10.1093/ismejo/wrag095 (PMC13184523; doi:10.1093/ismejo/wrag095)
Supplement: Yang_J_et_al_ms1_Suppl_figures_260408_wrag095 [file yang_j_et_al_ms1_suppl_figures_260408_wrag095.pdf]

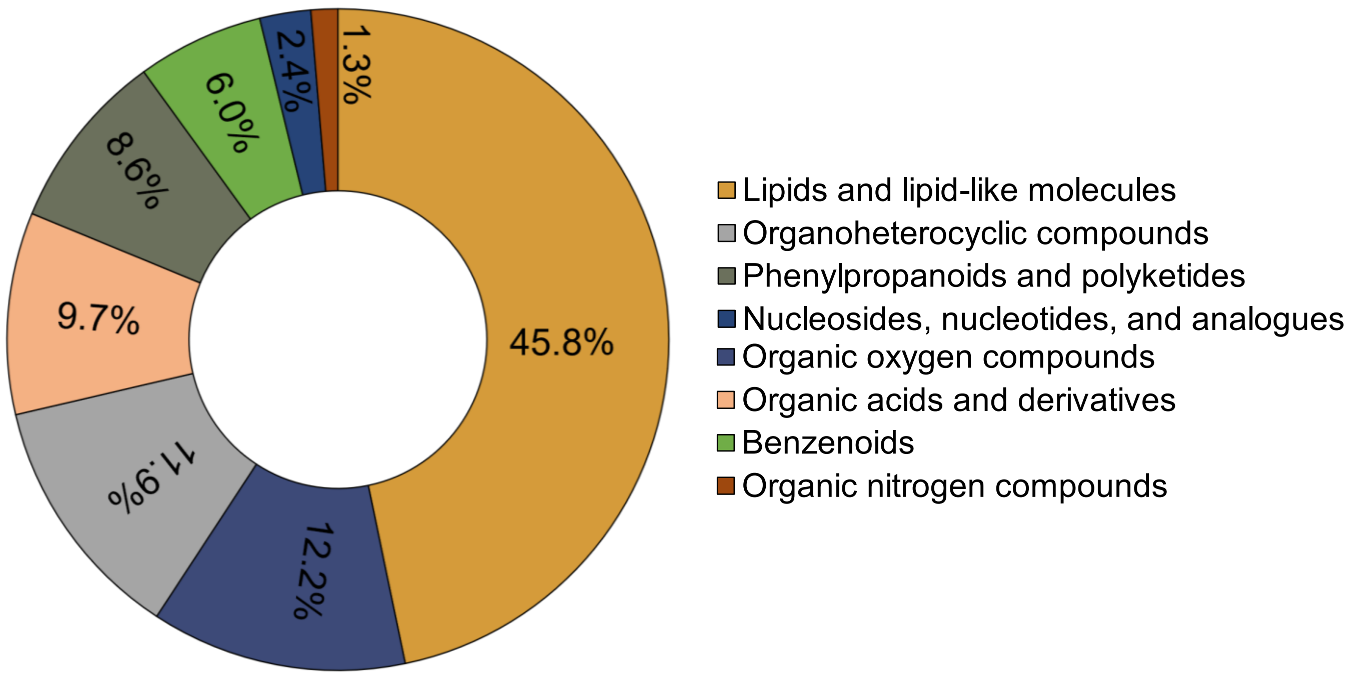

Fig. S1 Chemical classification of identified metabolites. The donut chart shows the percentage distribution of identified metabolites across different chemical classes. Each colored segment represents one chemical class, and the size of each segment indicates the proportion (%) of metabolites assigned to that class.

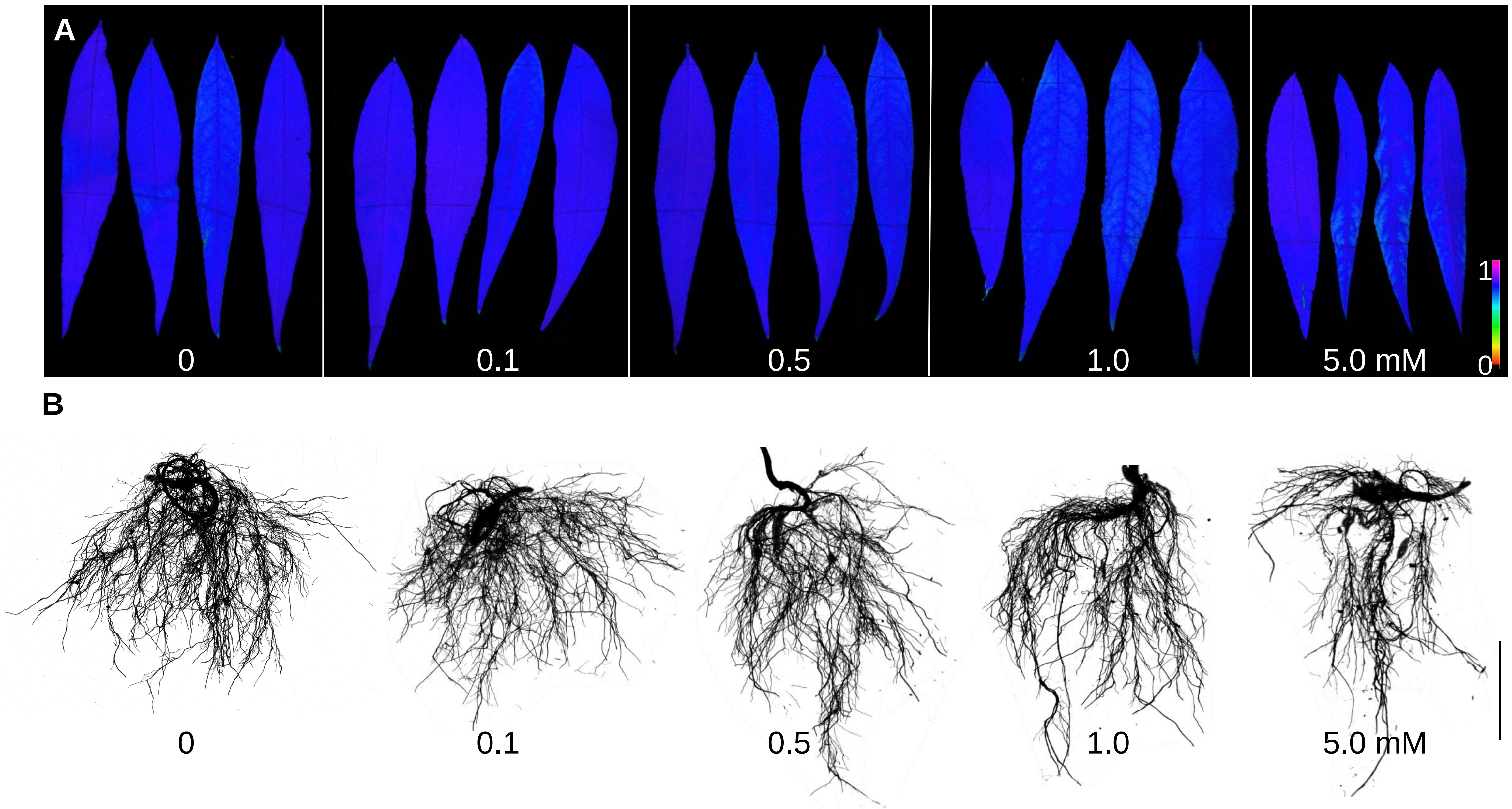

Fig. S2 Effects of different concentrations of amygdalin on the maximum PSII quantum yield in leaves and root system architecture. **A** False-color images of *Fv/Fm* values under different amygdalin concentrations (0, 0.1, 0.5, 1.0, and 5.0 mM), with the scale ranging from 0 (black) to 1.0 (purple). **B** Representative root morphology images acquired using a root scanner and analyzed with WinRHIZO software, scale = 5 cm.

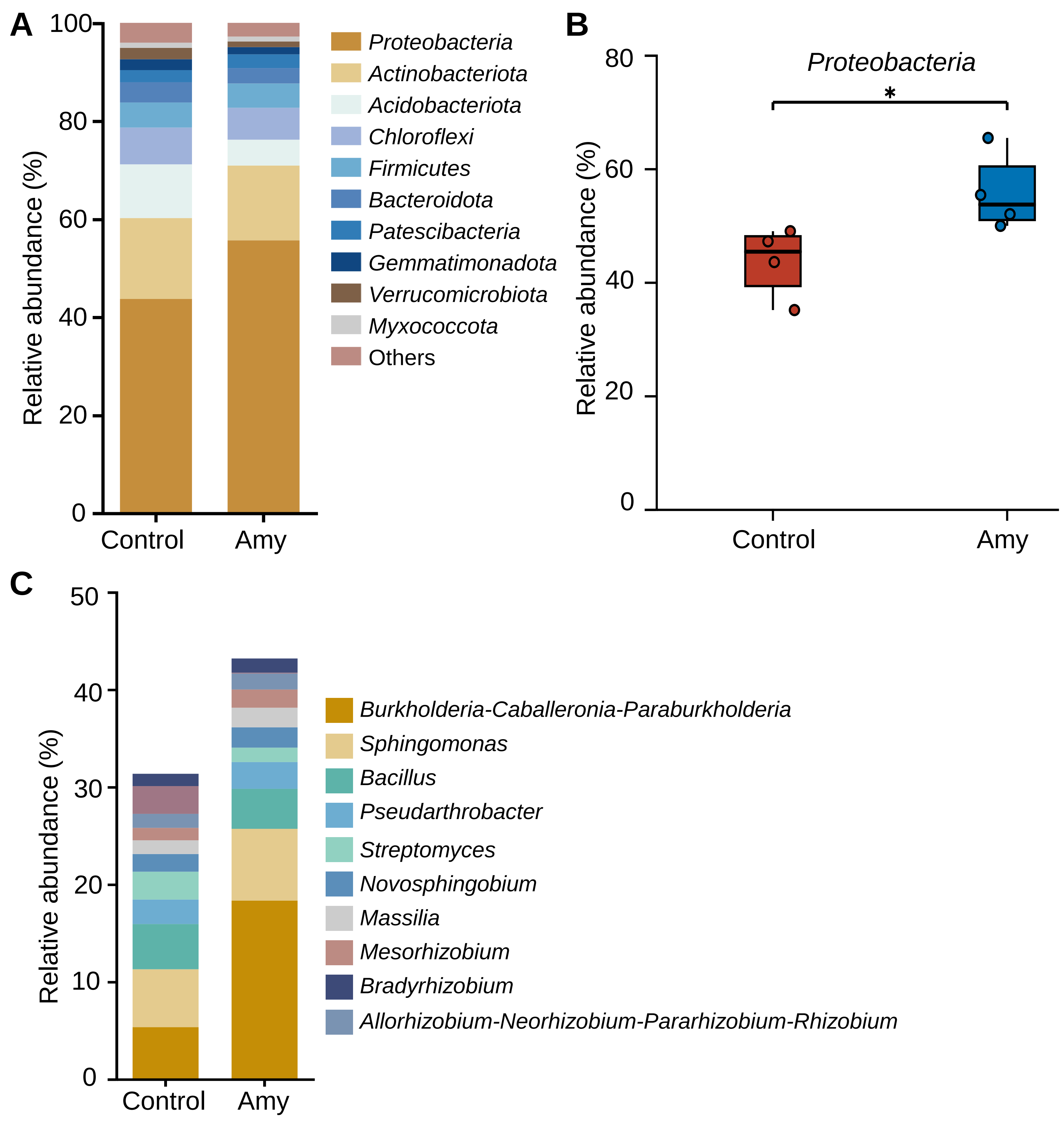

Fig. S3 Bacterial community composition shifts in the rhizosphere of peach seedlings in response to exogenous amygdalin (Amy) treatment. **A** Relative abundance of bacterial ASVs (≥ 1%) at the phylum level. **B** Relative abundance of *Proteobacteria* in the rhizosphere soil under control and amygdalin-amended conditions. Asterisk indicates statistical significance using a two-sided Student's *t*-test (*$P \leq 0.05$). **C** Taxonomic profile of bacterial ASVs at the genus level.

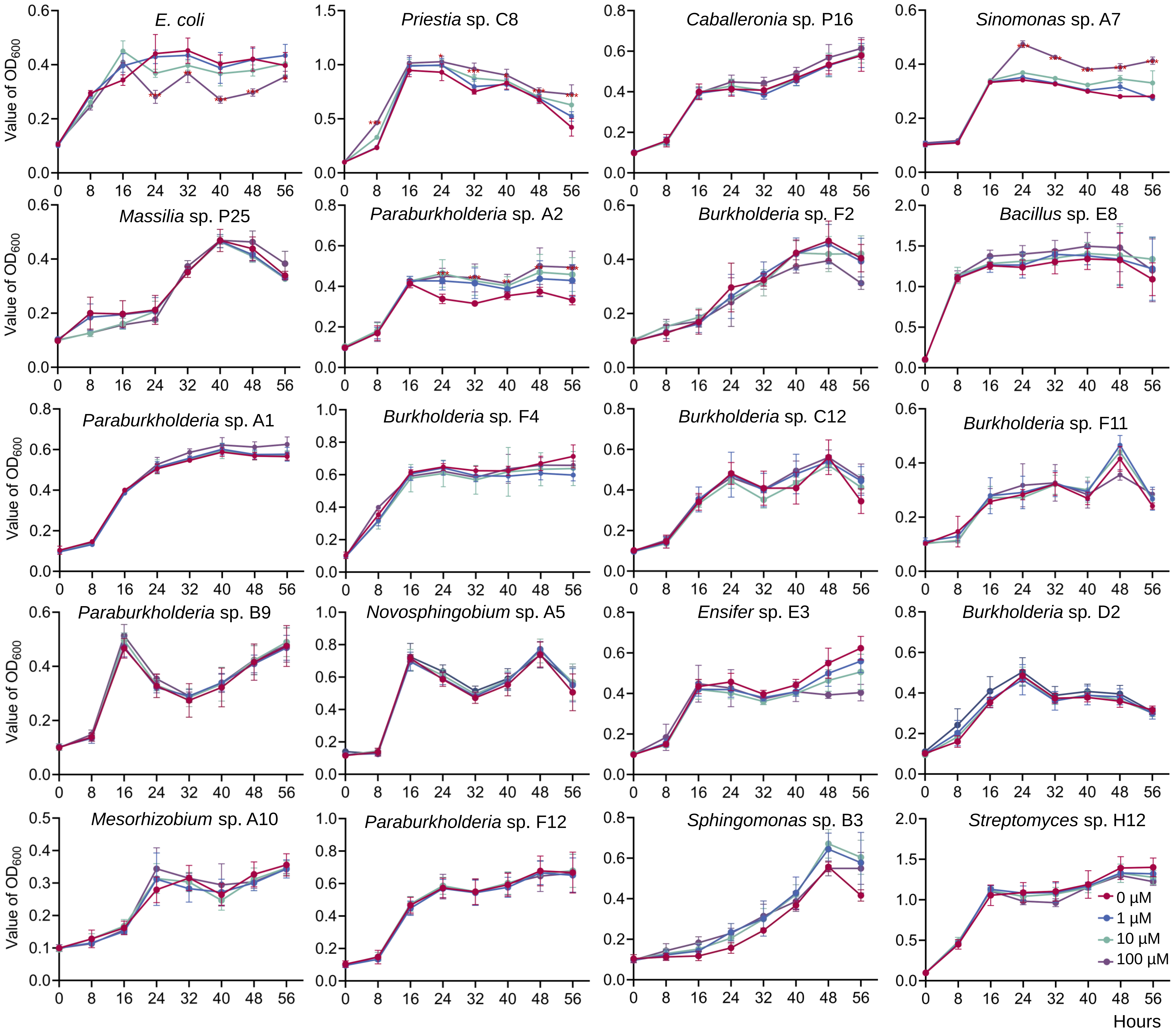

Fig. S4 The effect of amygdalin on bacterial growth was assessed by adding 10 μL of amygdalin stock solutions (0, 0.01, 0.1, or 1 mM) to 90 μL of 1/10 TSB medium inoculated with standardized cell suspensions of selected rhizobacterial strains, resulting in final concentrations of 0, 1, 10, and 100 μM, respectively. Data are presented as means ± SD (n = 7). *P < 0.05, **P < 0.01, ***P < 0.001 indicate statistically significant differences compared with the control group (amygdalin concentration = 0).

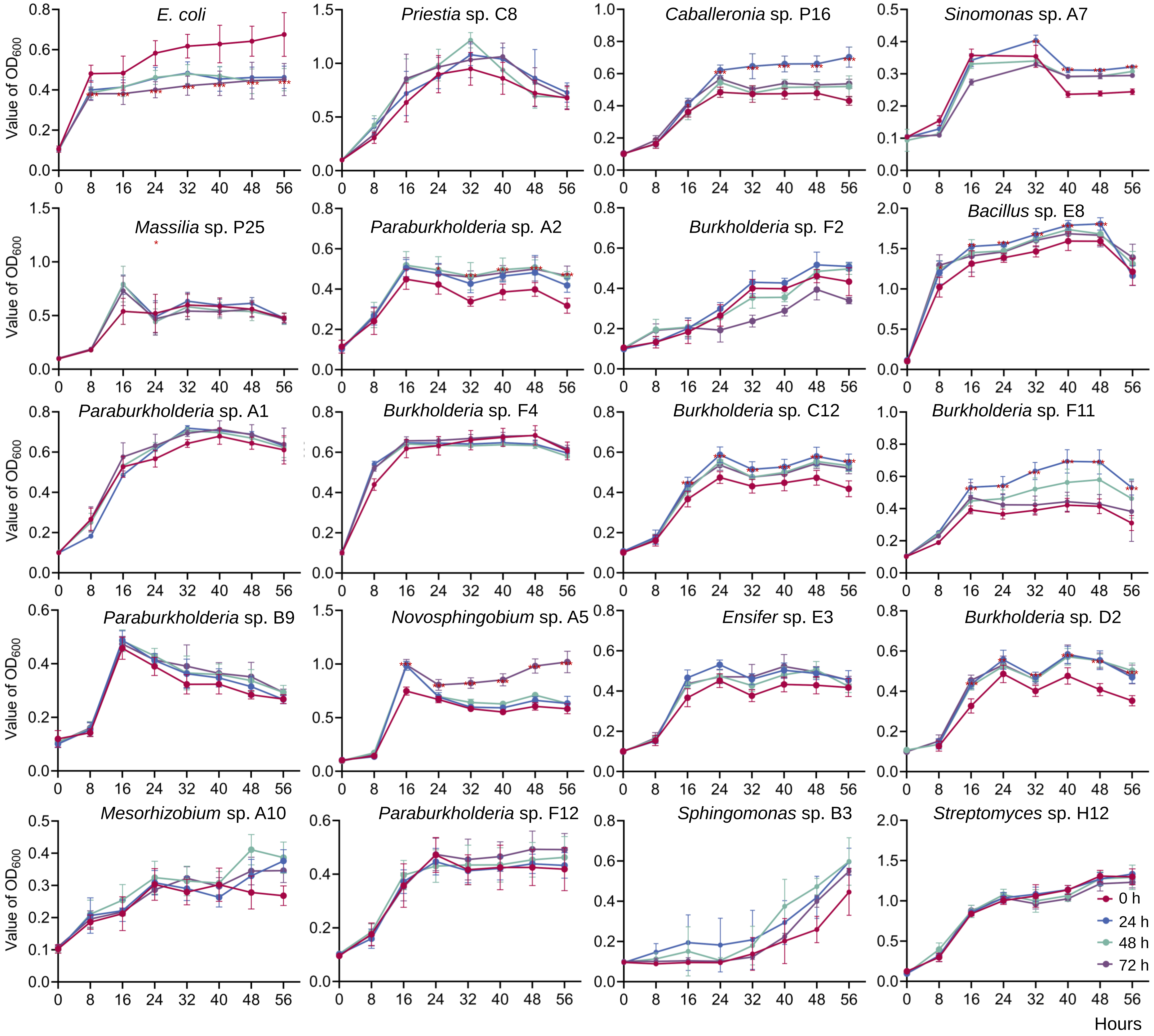

Fig. S5 Effects of soil-metabolized amygdalin products on the growth of peach rhizosphere bacteria. Bacterial growth was assessed by adding 10 µL of metabolite solution obtained after incubating 1 mM amygdalin with a PBS suspension containing peach rhizosphere soil for 24, 48, or 72 h to 90 µL of 1/10 TSB medium inoculated with standardized cell suspensions of selected rhizobacterial strains. The control group (0) received an equal volume of the corresponding metabolite-free control solution. Data are presented as means ± SD (n = 7). *P < 0.05, **P < 0.01, ***P < 0.001 indicate statistically significant differences compared with the control group.

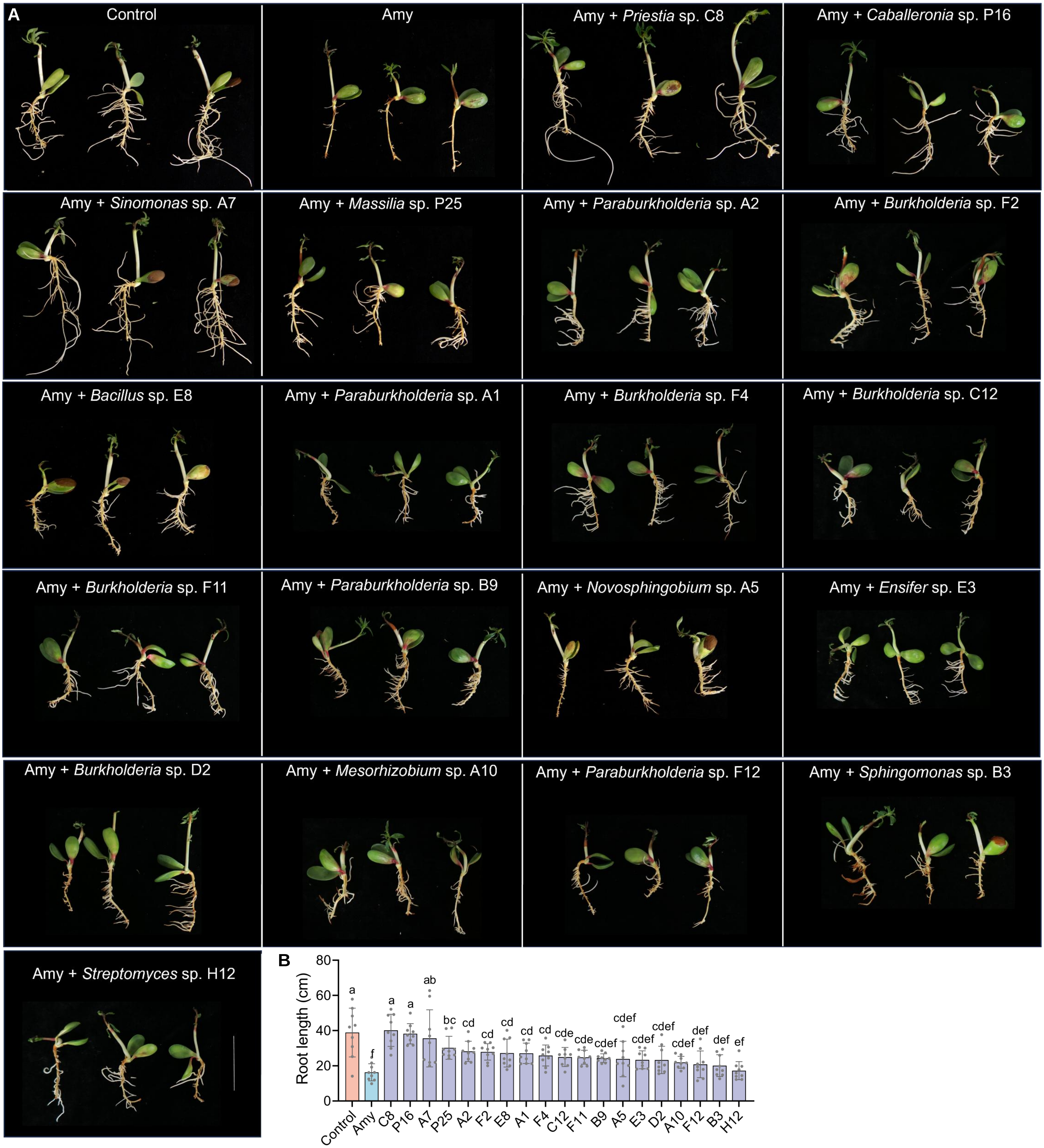

Fig. S6 Evaluation of the growth-promoting effects of 19 bacterial isolates on peach seedlings under amygdalin (Amy) stress. **A** Phenotypic responses of peach seedlings to bacterial inoculation in the presence of amygdalin. Scale bar = 5 cm. **B** Quantification of root length of peach seedlings. Data are presented as means ± SD (n = 9). Different letters indicate statistically significant differences at P < 0.05 based on Duncan's multiple range test.

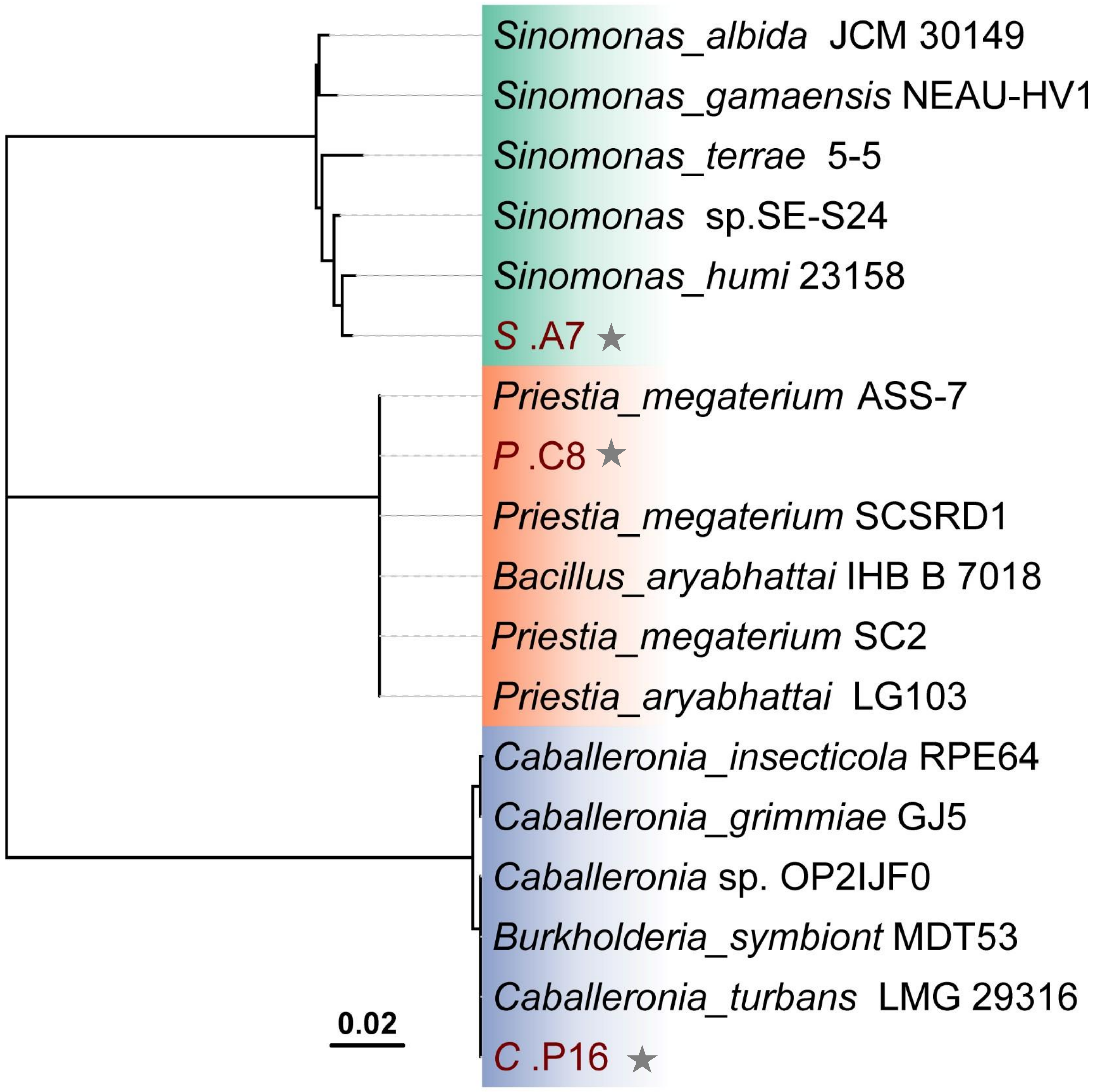

Fig. S7 Molecular identification of bacterial strains effective in alleviating amygdalin-induced stress in peach seedlings. A neighbor-joining phylogenetic tree based on 16S rRNA gene sequences was constructed in MEGA7 to show the phylogenetic positions of strains A7, C8, and P16 relative to closely related reference taxa. Strain A7 was most closely related to *Sinomonas humi*, strain C8 to *Priestia megaterium*, and strain P16 to *Caballeronia turbans*. The isolated strains are marked with asterisks.

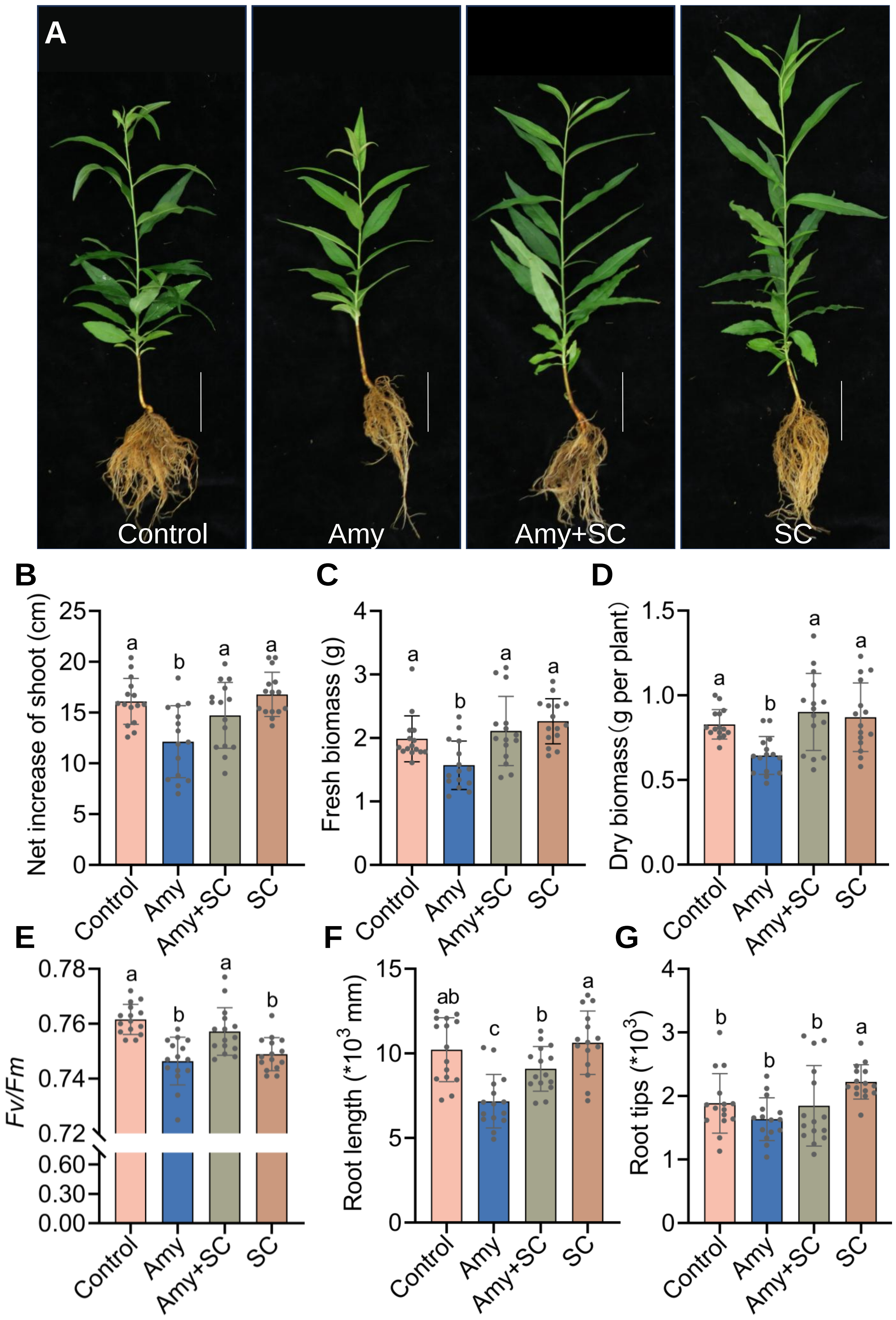

Fig. S8 Growth-promoting effects of synthetic community (SC) on peach seedlings grown in non-sterilized soil after 30 d of repeated amygdalin treatment (30 mL of 1 mM every 5 d). **A** Phenotypes of peach seedlings treated with amygdalin and inoculated with SC (Amy+SC), uninoculated seedlings under amygdalin (Amy) treatment, SC-inoculated seedlings alone (SC), plus uninoculated and untreated seedlings (Control). Scale bar = 5 cm. **B–G** Growth and physiological performance of peach seedlings: net shoot elongation (**B**), shoot fresh weight (**C**), plant dry biomass (**D**), maximum PSII efficiency *Fv*/*Fm* (**E**), root length (**F**), and number of root tips (**G**). Data represent means ± SD (n = 15). Different letters on top of bars indicate significant differences between different treatments ($P < 0.05$).

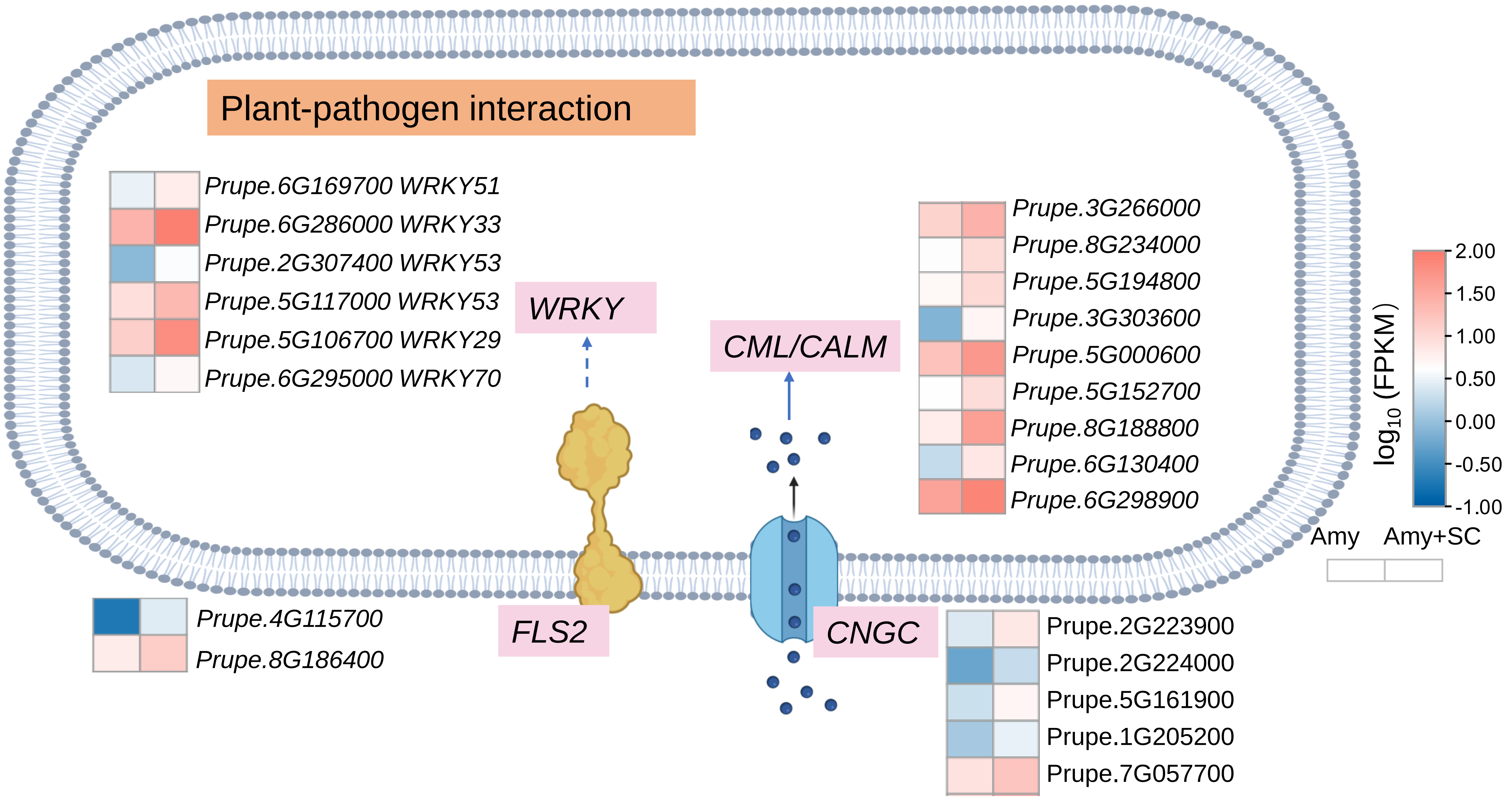

Fig. S9 Plant-pathogen interaction pathway responses in roots of amygdalin-stressed peach seedlings without (Amy) or with (Amy+SC) synthetic community inoculation. Differentially expressed genes (DEGs) were identified using DESeq2 (*FDR* < 0.05; |$\log_2$ fold change| > 1; *n* = 3 biological replicates). Heatmaps of DEGs, displaying $\log_{10}$ (FPKM) values, were generated using TBtools. Fig. 1F shows the experimental scheme.

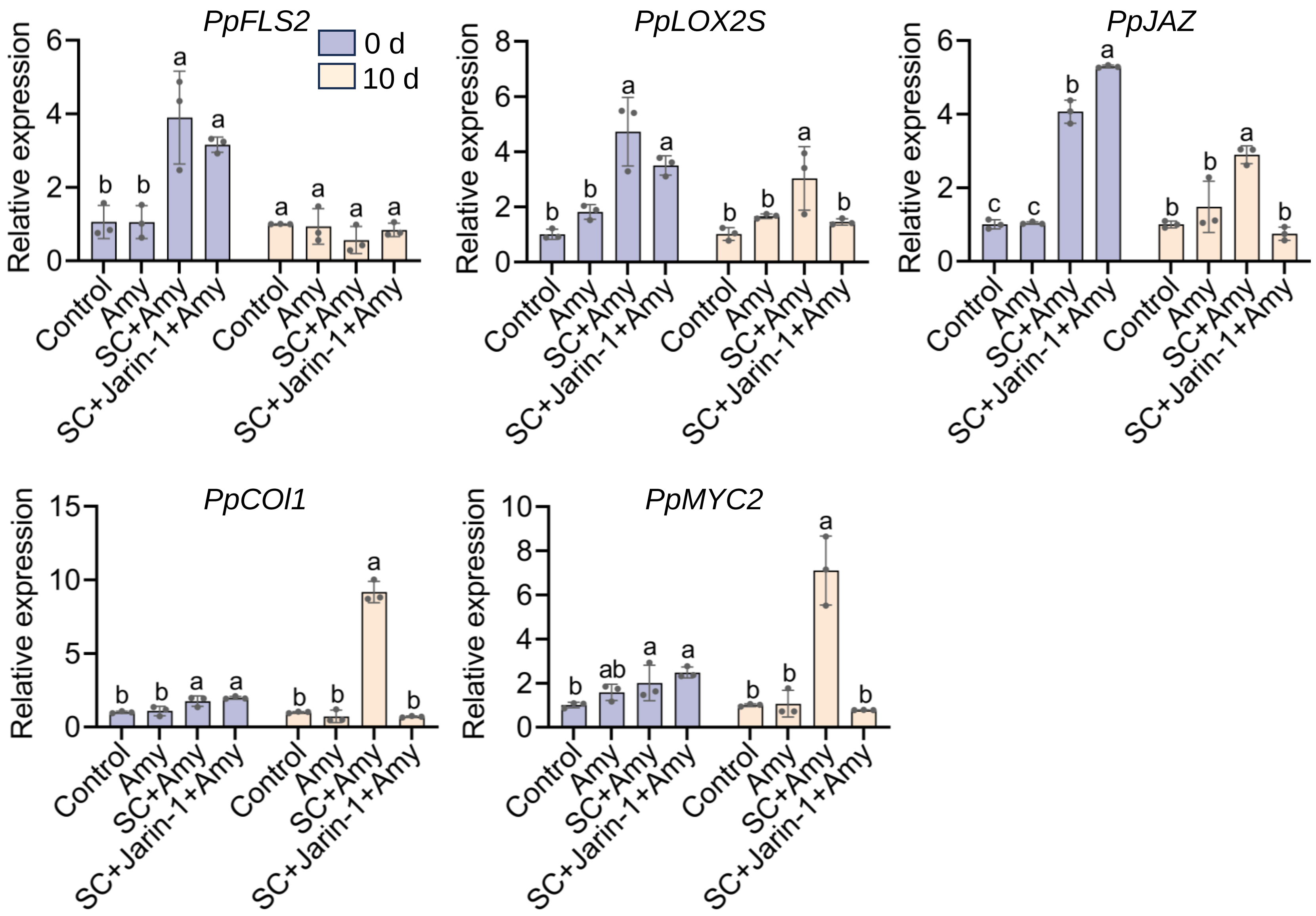

Fig. S10 Relative transcript abundance of the pattern recognition receptor gene *PpFLS2* (*FLAGELLIN SENSING 2*), the JA biosynthetic gene *PpLOX2S* (*LINOLEATE 13S-LIPOXYGENASE*), and the JA signaling genes *PpCOI1* (*CORONATINE-INSENSITIVE 1*), *PpJAZ* (*JASMONATE ZIM-DOMAIN*), and *PpMYC2* (transcription factor MYC2) in peach seedling roots in response to synthetic community (SC) inoculation and treatment with the JA-Ile biosynthesis inhibitor Jarin-1 under hydroponic conditions. Seedlings were assigned to four treatments: control, 0.5 mM amygdalin (Amy), SC inoculation for 2 h followed by 3 d of cultivation in Hoagland solution before 0.5 mM amygdalin treatment (Amy+SC), and SC inoculation for 2 h followed by 3 d of cultivation in Hoagland solution, 2 h of pretreatment with 10 μM Jarin-1, and subsequent 0.5 mM amygdalin treatment (Amy+SC+Jarin-1). Samples were collected at 0 and 10 d. At 0 d, samples were collected 3 d after SC inoculation before Jarin-1 or amygdalin treatment. At 10 d, samples were collected after 10 d of amygdalin treatment. Transcript levels were normalized to *PpTEF2* (*TRANSLATIONAL ELONGATION FACTOR 2*) and expressed relative to the untreated control. Data represent means ± SD of three biological replicates. Fig. 1H shows the experimental scheme.

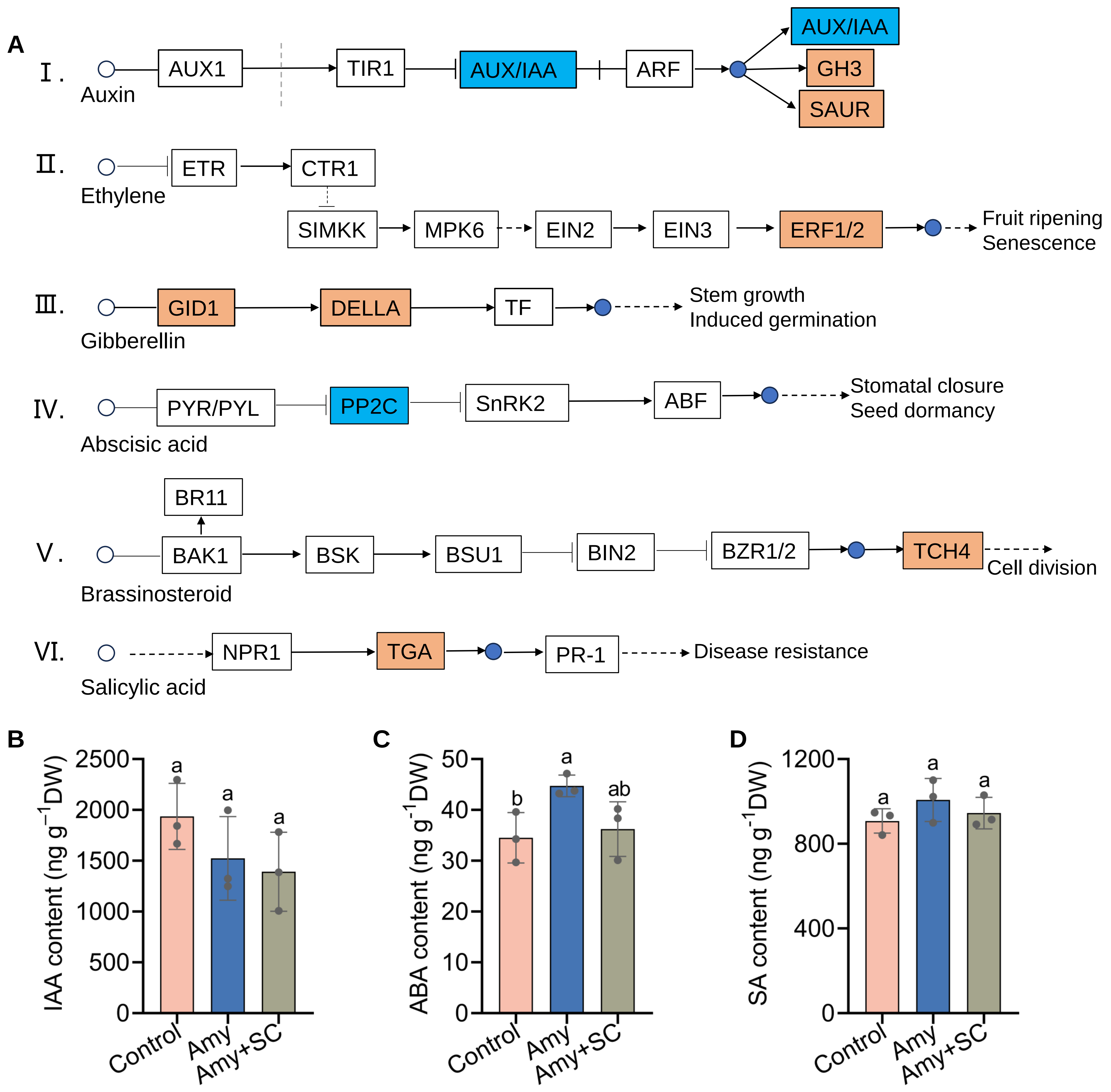

Fig. S11 Hormone-related transcriptomic and metabolomic analyses in peach roots under amygdalin treatment with or without SC inoculation. **A** Genes involved in auxin (IAA), ethylene (ET), gibberellin (GA), abscisic acid (ABA), brassinosteroid (BR), and salicylic acid (SA) signaling pathways are shown. Orange indicates Amy+SC significant upregulation, and blue indicates significant downregulation relative to the Amy. **B–D** IAA (B), ABA (C), and SA (D) contents in peach roots under the indicated treatments after 7 d. Data are shown as means ± SD (*n* = 3 biological replicates). Different letters indicate significant differences among treatments at *P* < 0.05. Fig. 1F shows the experimental scheme.
